# Hierarchically encapsulating enzymes with multi-shelled metal-organic frameworks for tandem biocatalytic reactions

**DOI:** 10.1038/s41467-022-27983-9

**Published:** 2022-01-13

**Authors:** Tiantian Man, Caixia Xu, Xiao-Yuan Liu, Dan Li, Chia-Kuang Tsung, Hao Pei, Ying Wan, Li Li

**Affiliations:** 1 Shanghai Key Laboratory of Green Chemistry and Chemical Processes, School of Chemistry and Molecular Engineering, East China Normal University, 500 Dongchuan Road, Shanghai, 200241 P. R. China; 2 School of Mechanical Engineering, Nanjing University of Science and Technology, Nanjing, 210094 P. R. China; 3 Hoffmann Institute of Advanced Materials, Shenzhen Polytechnic, 7098 Liuxian Boulevard, Nanshan District, Shenzhen, 518055 P. R. China; 4 Department of Chemistry, Merkert Chemistry Center, Boston College, 2609 Beacon Street, Chestnut Hill, MA 02467 USA

**Keywords:** Metal-organic frameworks, Biocatalysis

## Abstract

Biocatalytic transformations in living organisms, such as multi-enzyme catalytic cascades, proceed in different cellular membrane-compartmentalized organelles with high efficiency. Nevertheless, it remains challenging to mimicking biocatalytic cascade processes in natural systems. Herein, we demonstrate that multi-shelled metal-organic frameworks (MOFs) can be used as a hierarchical scaffold to spatially organize enzymes on nanoscale to enhance cascade catalytic efficiency. Encapsulating multi-enzymes with multi-shelled MOFs by epitaxial shell-by-shell overgrowth leads to 5.8~13.5-fold enhancements in catalytic efficiencies compared with free enzymes in solution. Importantly, multi-shelled MOFs can act as a multi-spatial-compartmental nanoreactor that allows physically compartmentalize multiple enzymes in a single MOF nanoparticle for operating incompatible tandem biocatalytic reaction in one pot. Additionally, we use nanoscale Fourier transform infrared (nano-FTIR) spectroscopy to resolve nanoscale heterogeneity of vibrational activity associated to enzymes encapsulated in multi-shelled MOFs. Furthermore, multi-shelled MOFs enable facile control of multi-enzyme positions according to specific tandem reaction routes, in which close positioning of enzyme-1-loaded and enzyme-2-loaded shells along the inner-to-outer shells could effectively facilitate mass transportation to promote efficient tandem biocatalytic reaction. This work is anticipated to shed new light on designing efficient multi-enzyme catalytic cascades to encourage applications in many chemical and pharmaceutical industrial processes.

## Abstract (web summary)

Mimicking multi-enzyme catalytic cascades in natural systems with spatial organization in confined structures is gaining increasing attention in the emerging field of systems chemistry. Here, the authors demonstrate that multi-shelled metal-organic frameworks can be used as a hierarchical scaffold to spatially organize enzymes on nanoscale to enhance cascade catalytic efficiency.

## Introduction

Tandem biocatalytic reactions represent a major class of chemical transformations that play important functions in biological signal transduction and metabolic pathways^1–4^. In biological systems, complex biocatalytic cascade processes occur in different cellular membrane-compartmentalized organelles to prevent unproductive cross-talk and malfunctions, thus producing biological products with unsurpassed efficiency^5–8^. Unsurprisingly, mimicking multi-enzyme catalytic cascades^9,10^ in natural systems with the spatial organization in confined structures is gaining increasing attention in the emerging field of systems chemistry^11–20^. The spatial organization of enzymes in metal-organic frameworks (MOFs) scaffolds provides a promising means to design multi-enzyme catalytic cascades, owing to MOFs’ unique advantages of tailorable crystalline and pore channels, large and accessible surface areas, and versatile framework compositions^21–29^. Several self-assembled MOF structures have been used to operate biocatalytic cascades. For example, MOF capsules assembled at Pickering emulsion^19,30–36^ and MOF chains linked by complementary peptides^37^, were manipulated to form distinguished spatial compartments to trigger multi-enzyme catalytic cascade. These systems immobilized different enzymes separately in MOF capsules or particles; however, reached limitations in catalytic efficiency due to slow transportations of intermediate products between compartments^19,37,38^. Hierarchically structured MOFs consisting of cavities with different sizes were also applied to a couple of multi-enzyme catalytic cascade^39,40^; nevertheless, these systems required stringent design and rigid synthesis condition. Recently, Willner’s group^41,42^ and other researchers^36,43–49^ (summarized in Supplementary Table 1) have reported direct encapsulation of multiple enzymes into single-spatial-compartmental MOF particles. The constructed biocatalytic cascades in the MOF nanoreactors show superior activities and operational stability compared with the catalytic cascades in the homogeneous aqueous phase. However, due to the inherent disadvantage of direct encapsulation, that is, enzymes were randomly distributed within MOF particles without control over spatial organization, these single-spatial-compartmental MOF particles cannot be used for the spatial confinement of incompatible and opposing reagents in one pot. It should be noted that incompatible (and competing) catalytic transformations, despite of their scientific and technological importance, have not previously been demonstrated in single MOF nanoparticles.

Meanwhile, mapping the spatial organization of enzymes in compartmental nanoreactors could provide an important perspective for regulating enzymatic interactions and developing cascade systems with high efficiency. Established techniques like Fourier transform infrared spectroscopy (FTIR) allows for nondestructive and label-free characterization, but it is limited for bulk sample analysis. Confocal microscopy has been a commonly used tool for verifying the confinement of enzymes in the single-spatial compartmental MOFs. However, its spatial resolution limited by diffraction (~λ/2) prevents further nanoscale enzyme studies. Nanoscale Fourier transform infrared (nano-FTIR) spectroscopy is a unique technique able to unveil the local chemical compositions with a spatial resolution down to ~10 nm. nano-FTIR spectroscopy is based on atomic force microscopy (AFM), where the metallic AFM tip is illuminated by the broadband infrared radiation. The tip acts as an antenna and concentrates the incident infrared field at the tip apex, yielding local infrared spectra with spatial resolution determined by the tip radius. On the above basis, nanoFTIR spectroscopy enables reliable probing and mapping of nanoscale chemical composition, and has been applied on the study of single membrane proteins^50^, multi-component polymer blends^51^, and coexisting nanoscale phases^52,53^, however has not previously been used to map the compartmentalized multi-enzymes.

Herein, we demonstrate that encapsulating multi-enzymes with multi-shelled MOFs by epitaxial shell-by-shell overgrowth could be used for enhancing catalytic efficiency in incompatible tandem biocatalytic reactions to overcome the aforementioned challenges. Using nano-FTIR spectroscopy, we chemically mapped the spatial organization of multi-enzymes in single multi-spatial-compartmental MOF particles with nanoscale resolution. The integration of compatible- and incompatible-enzyme cascade in multi-shelled MOFs leads to 5.8–13.5-fold enhancements in catalytic efficiencies compared with free enzymes in solution. Importantly, multi-shelled MOFs can act as a multi-spatial-compartmental nanoreactor that allows physically compartmentalize multiple enzymes in a single MOF nanoparticle for operating incompatible tandem biocatalytic reaction in one pot. Additionally, multi-shelled MOFs enable position control of multi-enzymes for facile regulation of catalytic efficiency. We show that close positioning of enzyme-1-loaded and enzyme-2-loaded shells along the inner-to-outer shells could facilitate tandem biocatalytic reactions with efficient mass transportation between spatially confined enzymes’ active sites. This work provides important insights into developing complex multi-spatial compartmental systems for multi-enzyme catalytic cascades that hold great promise in many industrial processes.

## Results

### Biocatalysis of a compatible-enzyme cascade encapsulated in multi-shelled ZIF-8

**Fig. 1:**
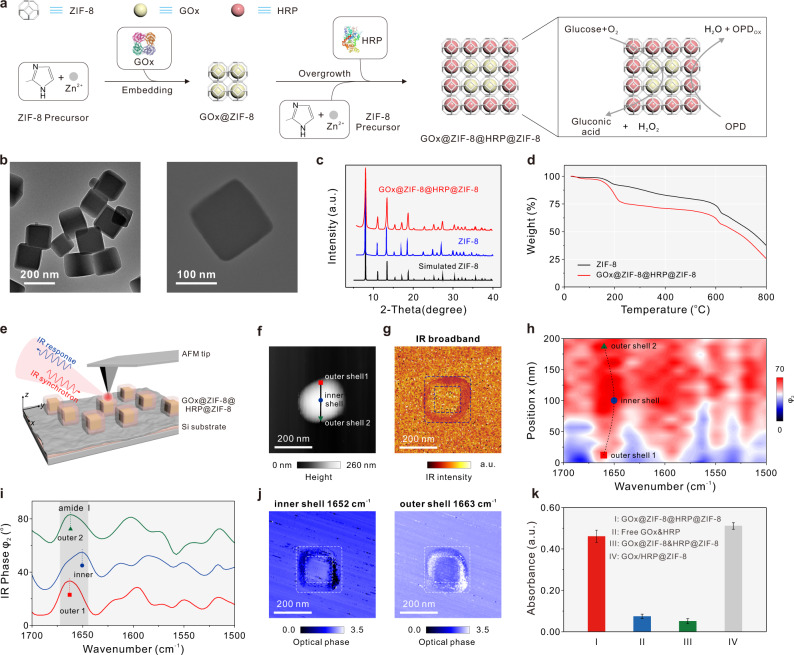
Operation of GOx-HRP cascade in multi-shelled ZIF-8 MOFs. **a** Tandem biocatalytic reaction driven by compatible enzymes (GOx and HRP) encapsulated in multi-shelled ZIF-8. **b** TEM image of as-synthesized GOx@ZIF-8@HRP@ZIF-8. **c** XRD pattern simulated from CIF file of ZIF-8 (black), XRD patterns of synthesized ZIF-8 (blue), and GOx@ZIF-8@HRP@ZIF-8 (red), respectively. **d** TGA curves of synthesized ZIF-8@ZIF-8 (black) and GOx@ZIF-8@HRP@ZIF-8 (red), respectively. Nanoscale heterogeneity in GOx@ZIF-8@HRP@ZIF-8 by nano-FTIR analysis: **e** Setup employing a mid-infrared laser continuum source or a tunable quantum cascade laser (QCL) for tip illumination. AFM mapping simultaneously retrieves nanoscale morphology and nano-FTIR spectra. Simultaneously recorded **f** AFM topography image and **g** IR broadband image. **h** nano-FTIR line scan along the solid arrow marked in panel **f** with a step size of ~10 nm, and 18 spectra were recorded. **i** Representative nano-FTIR point spectra of the inner shell (inner) and outer shell (outer 1 and outer 2) of the particle, marked by respective colored symbols. **j** Infrared phase images recorded at inner-shell 1652 cm^−1^ and outer-shell 1663 cm^−1^ of an individual particle. **k** Catalytic efficiencies in GOx@ZIF-8@HRP@ZIF-8, free GOx & HRP, GOx@ZIF-8 & HRP@ZIF-8, and GOx/HRP@ZIF-8 systems. In all experiments, the concentrations of GOx and HRP used were 4.71 and 14.4 μg mL^−1^. The error bars represent standard deviation based on three measurements.

Glucose oxidase (GOx) and horseradish peroxidase (HRP) were selected as model compatible enzymes for performing tandem biocatalytic reaction. As illustrated in Fig. 1a, in the first step, GOx was embedded within zeolitic imidazolate framework-8 (ZIF-8) via a de novo approach^54,55^. The resulting GOx@ZIF-8 was then used as cores to seed the overgrowth of the ZIF-8 shell in the second step. The HRP was similarly fixed into the second shell, resulting in the confinement of compatible enzymes in multi-shelled ZIF-8 (denoted as GOx@ZIF-8@HRP@ZIF-8). The obtained product showed different colors from ZIF-8 because of loaded enzymes in MOF scaffolds (Supplementary Fig. 1a–c). TEM imaging showed that both GOx@ZIF-8 and GOx@ZIF-8@HRP@ZIF-8 grew into cubic-shaped nanoparticles (Fig. 1b and Supplementary Fig. 2) and the average particle sizes were increased from ~80 ± 9 nm (ZIF-8), ~120 ± 15 nm (GOx@ZIF-8), to ~180 ± 20 nm (GOx@ZIF-8@HRP@ZIF-8) with enzymes encapsulated into different ZIF-8 shells (the corresponding particle size distribution based on dynamic light scattering (DLS) characterization is displayed in Supplementary Fig. 3). The nitrogen-sorption measurement was then conducted, and GOx@ZIF-8@HRP@ZIF-8 displayed type-I isotherms with a surface area of 938.04 m^2^g^−1^ from corresponding Brunauer-Emmett-Teller (BET) calculations, indicating that enzyme incorporation did not affect the MOF micropore structure^31^ (Supplementary Fig. 4). The crystallinity of the ZIF-8 and GOx@ZIF-8@HRP@ZIF-8 was examined by X-ray diffraction (XRD). The well-defined peaks indicated the good crystallinity, and both XRD spectra closely matched the simulated pattern, corresponding to the space group *I-43m* (Fig. 1c). The encapsulation efficiencies of GOx and HRP in the ZIF-8 were characterized by fluorescent spectrophotometry using calibration curves for the different dye-labeled enzymes^41,56^, and determined to be 81.34 ± 1.04% and 49.65 ± 1.22%, respectively (Supplementary Fig. 5 and Supplementary Table 2). The loadings of GOx and HRP in the ZIF-8 were 70.72 and 215.9 μg mg^−1^, which were found to be correlated to the weight loss detected in the thermal gravimetric analysis (TGA), confirming the presence of enzymes in the composite (Fig. 1d, see Supplementary Fig. 6 and Supplementary Table 3 for detailed discussion). Further insight into the confinement of enzymes was achieved through FTIR characterization (Supplementary Fig. 7). The bands centered at 1583 cm^−1^ and 1660 cm^−1^ of ZIF-8 generally appear coupled and could be assigned to N–H bending, C–N and C–C stretching vibrations of 2-methyl imidazole (HmIM) ring^57^. The characteristic band at 1583 cm^−1^ was also present in the GOx@ZIF-8@HRP@ZIF-8, suggesting the coordinating environment of HmIM in GOx@ZIF-8@HRP@ZIF-8 was consistent with that in ZIF-8^57^; while the weak band at 1660 cm^−1^ was superimposed by a much stronger absorption band of amide-I stretch in GOx@ZIF-8@HRP@ZIF-8. On the other hand, the FTIR spectra revealed a difference between confined and freestanding enzymes. Confined enzymes exhibit a blue shift of the amide-I stretch (1668 cm^−1^) compared to free GOx (1641 cm^−1^) and free HRP (1651 cm^−1^), indicating the enzyme-ZIF-8 interaction that arises from the coordination between Zn cations and carbonyl group of enzymes^31,57^. Despite that the infrared spectral analysis provides valuable insights, the diffraction-limited resolution of FTIR prevents visualization of the nanoscale distribution of enzymes in ZIF-8 framework. Next, as a first application example, we introduced nano-FTIR spectroscopy and demonstrated nanoscale mapping of the spatial distribution of multi-enzymes in individual multi-shelled ZIF-8 particles (Fig. 1e, see Supplementary Fig. 8 for further details). Figure 1f, g show simultaneously recorded AFM topography and IR broadband images of a single GOx@ZIF-8@HRP@ZIF-8 particle. The IR broadband image clearly reveals heterogeneity in vibrational activity between the inner-shell and outer-shell. To further analyze the nanoscale chemical composition, we recorded a nano-FTIR line scan along the solid arrow in the topography image across the particle (Fig. 1h, i). The spectrum recorded in the inner-shell region presents characteristic amide I at ~1652 cm^−1^, and shifted to ~1663 cm^−1^ in the out-shell region, which is consistent with the observation in far-field FTIR absorbance spectra (Supplementary Fig. 7). In a control experiment, we carried out nano-FTIR spectroscopy on single-enzyme-loaded ZIF-8 samples (GOx@ZIF-8 and Pro@ZIF-8, Supplementary Fig. 9), which revealed homogeneous IR broadband response. The representative point-spectra collected in single-enzyme-loaded ZIF-8 corroborate with those collected in the respective regions in multi-shelled ZIF-8, indicating the differences in coordinating environment for different enzymes^57^. Further nano-FTIR spectroscopy on pure ZIF-8 (Supplementary Fig. 10) also reveals the characteristic spectral feature at ~1587 cm^−1^, which agrees well with the FTIR spectrum (Supplementary Fig. 7); and does not reveal clear presence of ~1660 cm^−1^ band, which could be attributed to its weak absorption and possible suppression in the nano-FTIR spectra. Thus, we could assign the characteristic spectral component in the frequency range of 1600–1700 cm^−1^ to amide-I stretch of immobilized enzymes. On the above basis, we then imaged this particle at two selected wavenumbers (inner-shell: 1652 cm^−1^, outer-shell: 1663 cm^−1^) corresponding to the maximum vibrational absorption of amide I in inner- and outer-shells. As shown in Fig. 1j, the chemical contrast between different shells can be clearly resolved. To sum up, all the aforementioned results confirm the successful confinement of two compatible enzymes in multi-shelled ZIF-8 MOFs.

The tandem biocatalytic reaction in multi-shelled ZIF-8 MOFs was then investigated using glucose as the substrate. The aerobic oxidation of glucose in the presence of GOx produces gluconic acid and H_2_O_2_, H_2_O_2_ then acts as an oxidizing substrate for HPR which catalyzes the oxidation of o-phenylenediamine (OPD) to form fluorescent 2,3-diaminophenazine (DAP)^58,59^. Control experiments revealed that pure ZIF-8 cannot trigger the biocatalytic cascade (Supplementary Fig. 11). As shown in Fig. 1k, GOx@ZIF-8@HRP@ZIF-8 showed ~5.8-fold and ~9.1-fold catalytic activity improvement than homogeneous diffusional mixture of free enzymes (free GOx&HRP) and unassembled single-enzyme-loaded ZIF-8 (GOx@ZIF-8&HRP@ZIF-8), respectively (For detailed comparison with previously reported systems^49,60,61^, please see Supplementary Table 1). As the molecular diameter of glucose with ~9 Å (Supplementary Fig. 12) is intermediate between the aperture (~3.4 Å) and pore (~11.6 Å) of ZIF-8^62^, we reason the mass transport is likely facilitated by the short-lived “open states” of the expanded apertures due to mobile HmIM linker and ubiquitous presence of defects involving dangling linker and linker vacancies^63–65^. The observed activity enhancement of multi-enzyme cascades in multi-shelled MOFs is in agreement with previous theoretical and experimental conclusions^66–70^. The intermediates following the designated diffusion pathway as confined by the pore channels, leading to longer intermediate dwelling time and ensuing increased intermediate local concentration in the vicinity of HRP site relative to the disorganized multi-enzyme system. Furthermore, we compared the catalytic activity with the single-spatial-compartmental ZIF-8 MOFs (i.e., GOx/HRP@ZIF-8). It is discovered that GOx@ZIF-8@HRP@ZIF-8 exhibited slightly decreased catalytic efficiency than GOx/HRP@ZIF-8 (Fig. 1k), which is similar to previous work^71,72^ that demonstrated close integration between GOx and HRP could facilitate in situ formed H_2_O_2_ to immediately react with adjoining HRP, thus minimizing diffusion and self-decomposition of H_2_O_2_. Nevertheless, it is worth noting that GOx@ZIF-8@HRP@ZIF-8 still yielded 90.2% catalytic efficiency of GOx/HRP@ZIF-8, indicating that there is insignificant mass transport resistance cross over compartment boundaries within multi-shelled ZIF-8^41^.

Moreover, control experiments excluded possible contributions from the surface adsorption of enzymes on multi-shelled ZIF-8 in the tandem biocatalytic reaction. As displayed in Supplementary Fig. 13, GOx@ZIF-8@HRP@ZIF-8 was treated with trypsin for 24 h at 37 °C, whereby any surface-adsorbed enzymes would be hydrolytically digested. The GOx@ZIF-8@HRP@ZIF-8 retained 94.1% of their original activity after treatment with trypsin, confirming that negligible enzymes were adsorbed on the surfaces of multi-shelled ZIF-8. In contrast, free GOx&HRP lost 82.2% of their original activity, exemplifying the shielding function of the MOF scaffold against digesting enzymes. Also, the leaching of enzymes from ZIF-8 was revealed to be negligible (Supplementary Fig. 14). In addition, compartmentalization enhances the long-term storage stability of enzymes. As displayed in Supplementary Fig. 15, after storing at 4 °C for 10 days, GOx@ZIF-8@HRP@ZIF-8 showed only a marginal loss of activity (6.04% of its original activity); whereas free GOx&HRP lost nearly 44.3% of their original activity. Both TEM and XRD characterizations (Supplementary Figs. 16–17) confirmed that GOx@ZIF-8@HRP@ZIF-8 maintained structural integrity and good crystallinity after 10 days storage and after catalytic reaction post-storage. To sum up, these results reveal that multi-shelled MOFs can act as effective multi-spatial-compartmental nanoreactors for designing multi-enzyme catalytic cascades.

### Biocatalysis of an incompatible-enzyme cascade encapsulated in multi-shelled hollow ZIF-8

**Fig. 2:**
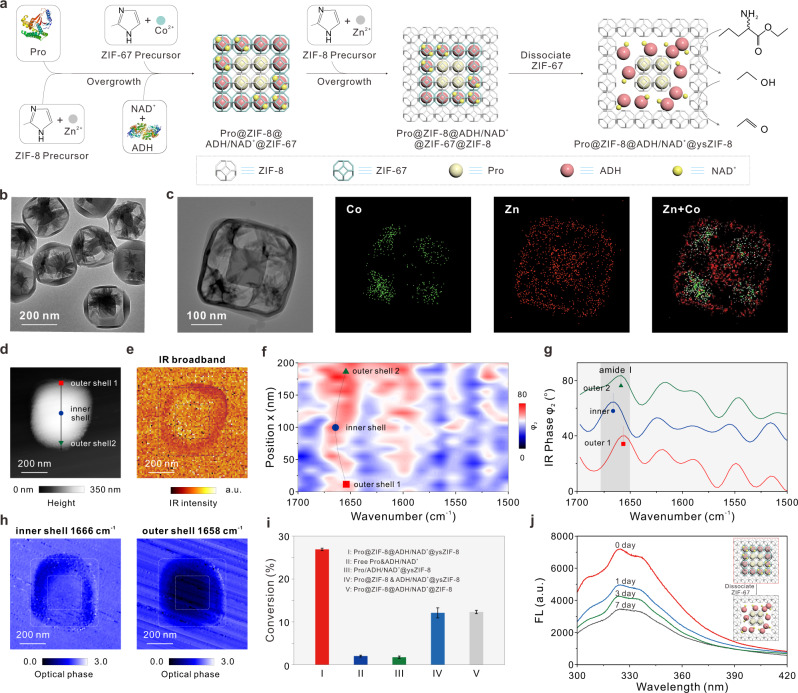
Operation of Pro-ADH/NAD^+^ cascade in multi-shelled hollow ZIF-8 MOFs. **a** Tandem biocatalytic reaction driven by incompatible enzymes and cofactor (Pro and ADH/NAD^+^) encapsulated in multi-shelled hollow ZIF-8. **b** TEM, **c** STEM and EDX mapping images of as-synthesized Pro@ZIF-8@ADH/NAD^+^@ysZIF-8. Nanoscale heterogeneity in Pro@ZIF-8@ADH/NAD^+^@ysZIF-8 by nano-FTIR analysis. simultaneously recorded **d** AFM topography image and **e** IR broadband image. **f** Nano-FTIR line scan along the solid arrow marked in panel d) with a step size of ~10 nm, and 20 spectra were recorded. **g** Representative nano-FTIR point spectra of the inner shell (inner) and outer shell (outer 1 and outer 2) of the particle, marked by respective colored symbols. Infrared phase images were recorded at **h** inner-shell 1666 cm^−1^ and outer-shell 1658 cm^−1^ of an individual particle. **i** Catalytic efficiencies in Pro@ZIF-8@ADH/NAD^+^ @ysZIF-8, free Pro & ADH/NAD^+^, Pro/ADH/NAD^+^@ysZIF-8, Pro@ZIF-8 & ADH/NAD^+^@ ysZIF-8, and Pro@ZIF-8@ADH/NAD^+^@ZIF-8 systems. **j** ADH emission spectrum in Pro@ZIF-8@ADH/NAD^+^@ZIF-67@ZIF-8 with different etching time of 0 day, 1 day, 3 day, 7 day (excitation wavelength: 280 nm, the diagram is the schematic illustration of association/dissociation of ADH/NAD^+^ cofactors encapsulated in solid and hollow MOFs). In all experiments, the concentrations of Pro, ADH, and NAD^+^ used were 5.35, 19.3, and 17.1 μg mL^−1^. The error bars represent standard deviation based on three measurements.

Having established multi-shelled MOFs as effective multi-spatial-compartmental nanoreactors, we next selected alkaline protease (Pro) and alcohol dehydrogenase (ADH) as model incompatible enzymes, to further substantiate the incompatible tandem biocatalytic reaction within single MOF particles in one pot. Pro is known as a digestive enzyme^18,19^, which has to be physically separated from ADH and cofactor NAD^+^ to prevent degradation and allow the cascade process to take place normally. NAD^+^-dependent ADH, which uses the interconversion of the NAD^+^/NADH redox couple to catalyze the oxidation of alcohols into corresponding aldehydes, is one of the most important biocatalysts for metabolic engineering, biosensors, and biofuel cells^73,74^. Note that we herein modified the protocol to fabricate multi-shelled hollow ZIF-8 MOFs to ensure the freedom of NAD^+^/NADH cofactors that operate diffusional in nature^41^. As illustrated in Fig. 2a, following a similar two-step process^55^, Pro and ADH/NAD^+^ were sequentially encapsulated within different shells of multi-shelled ZIF-8@ZIF-67 MOFs. Followed by epitaxial shell-by-shell overgrowth of ZIF-8, Pro@ZIF-8@ADH/NAD^+^@ZIF-67@ZIF-8 was obtained. Then, we dissociated the ZIF-67 to produce a hollow yolk-shell structure^55,75^ (Fig. 2b). The Pro originally embedded in ZIF-8 shells are still fixed in the ZIF-8 shells, and the ADH/NAD^+^ originally embedded in the ZIF-67 are released and confined in hollow cavities between ZIF-8 shells, which is termed as Pro@ZIF-8@ADH/NAD^+^@ysZIF-8. The obtained Pro@ZIF-8@ADH/NAD^+^@ysZIF-8 exhibited purple color due to the existence of cobalt hydroxide (Supplementary Fig. 18). TEM imaging showed that Pro@ZIF-8 and Pro@ZIF-8@ADH/NAD^+^@ysZIF-8 exhibited average particle sizes of ~140 ± 33 nm (Pro@ZIF-8) and ~280 ± 80 nm (Pro@ZIF-8@ADH/NAD^+^@ysZIF-8) (Fig. 2b and Supplementary Fig. 19. The corresponding particle size distribution based on DLS characterization is displayed in Supplementary Fig. 20), respectively. TEM-assisted energy-dispersive X-ray (EDX) was used to examine the yolk-shell structure after the dissociation. EDX elemental mapping shows that zinc is uniformly distributed in its own shells while cobalt ions dissociate from ZIF-67 and generate sheet-like amorphous cobalt hydroxides (Fig. 2c and Supplementary Fig. 21), which can function as a support to hold the cavity space between ZIF-8 shells^76^. Note that the hollowing property can be controlled by varying the dissociation degree and the enzyme leaching was not observed over the time course, as demonstrated by a time-dependent TEM study and fluorescent characterization (Supplementary Figs. 22 and 23). Next, we carried out a nitrogen-sorption measurement (Supplementary Fig. 24), and as-synthesized Pro@ZIF-8@ADH/NAD^+^@ysZIF-8 displayed type-I isotherms with a surface area of 1609.76 m^2^g^−1^ from BET calculations. A typical desorption hysteresis loop was observed in the pressure range of 0.48 to 1, confirming the formation of mesostructured voids between the ZIF-8 shells^55^. The crystallinities of the ZIF-8, intermediate Pro@ZIF-8@ADH/NAD^+^@ZIF-67@ZIF-8, and Pro@ZIF-8@ADH/NAD^+^@ysZIF-8 were then examined by XRD. As displayed in Supplementary Fig. 25, due to the fact that ZIF-8 and ZIF-67 are isostructural, XRD patterns are nearly identical for all three samples and correspond to the space group *I-43m*^77^. Note that no additional reflections corresponding to other crystalline Co species were observed, which is similar to previous work^76,78^. The encapsulation efficiencies of Pro and ADH/NAD^+^ in the ZIF-8 were characterized by fluorescent spectrophotometry using calibration curves for the RhB-labeled Pro, FITC-labeled ADH, and coumarin-labeled NAD^+^ (Supplementary Fig. 26 and Supplementary Table 2), and determined to be 77.38 ± 1.37%, 55.20%±2.91%, and 48.90 ± 3.77%, respectively. The loadings of Pro, ADH, and NAD^+^ were 53.49, 193.0, and 170.9 μg mg^−1^, which agreed with the weight loss measured in TGA (see Supplementary Fig. 27 and Supplementary Table 4 for detailed discussion). Moreover, the FTIR spectra confirmed the presence of the C = O stretching bands of amide I from the loaded enzymes, which were absent in the ZIF-8 (Supplementary Fig. 28). Further analysis shows that the amide vibrational mode (mainly from C = O stretching) is shifted towards high wavenumbers after encapsulation into multi-shelled ZIF-8 (before, Pro: 1653 cm^−1^, ADH: 1644 cm^−1^; after, Pro@ZIF-8@ADH/NAD^+^@ysZIF-8: 1671 cm^−1^). This characteristic spectral feature was consistent with that in GOx@ZIF-8@HRP@ZIF-8 and similarly observed in previous work^36,58,79^, which can be attributed to chemical interaction between confined enzymes and ZIF-8 framework due to the coordination between Zn cations and carbonyl group of enzymes^36^. Next, we conducted nano-FTIR spectroscopy in Pro@ZIF-8@ADH/NAD^+^@ysZIF-8. Figure 2d, e show simultaneously recorded AFM topography and IR broadband images of a single Pro@ZIF-8@ADH/NAD^+^@ysZIF-8 particle. The IR broadband image maps heterogeneity in vibrational response between the inner-shell and outer-shell, which is in clear contrast to the homogeneous response revealed in single-enzyme-loaded ZIF-8 samples (Pro@ZIF-8 and ADH/NAD^+^@ysZIF-8, Supplementary Fig. 29). The nano-FTIR line scan across the particle (Fig. 2f, g) shows that spectra collected in the inner-shell region presents characteristic amide I at 1666 cm^−1^, and red-shifted to 1658 cm^−1^ in the out-shell region, where is consistent with the observation in far-field FTIR absorbance spectra (Supplementary Fig. 28) and single-enzyme-loaded ZIF-8 samples (Pro@ZIF-8 and ADH/NAD^+^@ZIF-8, Supplementary Fig. 29). The spatial heterogeneity in Pro@ZIF-8@ADH/NAD^+^@ysZIF-8 particle can also be clearly recognized in the optical phase images recorded at two selected wavenumbers (Fig. 2h, inner-shell: 1666 cm^−1^, outer-shell: 1658 cm^−1^), which were recorded simultaneously to topography. Taken together, these results confirm the successful confinement of two incompatible enzymes and cofactor in multi-shelled hollow ZIF-8 MOFs.

In the presence of l-norvaline ethyl ester hydrochloride, the tandem biocatalytic reaction is activated. In this process, Pro catalyzes the conversion of the ester bond in l-norvaline ethyl ester hydrochloride to ethanol, and ADH catalyzes the oxidation of ethanol to acetaldehyde, with the concomitant reduction of NAD^+^ to NADH (Supplementary Fig. 30)^73,74,80,81^. The catalytic activity was characterized using gas chromatography (Supplementary Fig. 31). Control experiments revealed that pure ZIF-8@ysZIF-8 and Pro@ZIF-8@ysZIF-8 cannot trigger the biocatalytic cascade (Supplementary Fig. 32). As displayed in Fig. 2i, Pro@ZIF-8@ADH/NAD^+^@ysZIF-8 exhibited 13.5-fold and 15.4-fold production yields of acetaldehyde compared with those in the free Pro & ADH/NAD^+^ and single-spatial-compartmental ZIF-8 (i.e., Pro/ADH/NAD^+^@ysZIF-8) systems in which the tandem reaction was severely inhibited due to the proteolytic degradation of other components. Additionally, the biocatalytic cascade in Pro@ZIF-8@ADH/NAD^+^@ysZIF-8 was ~2.3-fold enhanced compared with homogenous diffusional mixture of unassembled single-enzyme-loaded MOFs (Pro@ZIF-8 & ADH/NAD^+^@ysZIF-8). Similar to the GOx@ZIF-8@HRP@ZIF-8, the molecular diameter of L-norvaline ethyl ester hydrochloride (Supplementary Fig. 12d) is ~7.2 Å, the molecular diffusion in the porous matrices of ZIF-8 could be likely facilitated by mobile linkers and missing-linker defects^63–65^. Overall, this result is in line with previous GOx-HRP cascade system (Fig. 1k), and further validates the advantage of constructing multi-spatial-compartmental MOFs for efficient operation of tandem biocatalytic reaction.

Next, we investigated the effect of hollowing process on catalytic activity of Pro-ADH/NAD^+^ to demonstrate the necessity of constructing a hollow architecture for NAD^+^-dependent enzyme cascades. As displayed in Fig. 2i and Supplementary Fig. 33, control experiments in the analogous MOFs without hollow nanocages (*i.e*., Pro@ZIF-8@ADH/NAD^+^@ZIF-8) exhibited a significant decrease (54.1%) in the production yield; and the catalytic efficiency gradually increased with decreased confined state of ADH-NAD^+^/NADH during the hollowing process. ADH relies on coenzyme dissociation and association that involves conformational changes and interconversion of NAD^+^/NADH redox couple to catalyze the oxidation of alcohol to aldehyde (see Supplementary Fig. 34 for detailed discussion on the mechanism of NAD^+^-dependent ADH)^82–84^. We then probed the interaction of NAD^+^/NADH with ADH in solid and hollow MOFs by monitoring the intrinsic tryptophan fluorescence in ADH during the hollowing process. The intrinsic tryptophan fluorescence in ADH could be quenched by NAD^+^/NADH binding, which is ascribed to conformational changes in ADH carrying the tryptopahn residue into a quenching environment^84^. As expected, we observed the progressive quenching during the hollowing process, suggesting that a lesser confined state facilitated more NAD^+^/NADH binding onto ADH active sites (Fig. 2j and Supplementary Fig. 35). On the other hand, we found that the catalytic activity was not affected by cobalt hydroxide (Supplementary Fig. 36). Despite that the lesser confined state benefits activity, we still kept the cobalt hydroxide support for the sake of balancing the trade-off between stability and activity.

Subsequently, we examined the long-term storage stability of incompatible enzymes encapsulated multi-shelled hollow ZIF-8 MOFs. As displayed in Supplementary Fig. 37, Pro@ZIF-8@ADH/NAD^+^@ysZIF-8 maintained structural integrity and catalytic activity (with only 14.1% loss of activity) after storing at 4 °C for 10 days, exemplifying the shielding function of the MOF scaffold. In contrast, free Pro & ADH/NAD^+^ lost catalytic activity nearly instantly due to the protease digestion. Both TEM and XRD characterization (Supplementary Figs. 38–39) confirmed that Pro@ZIF-8@ADH/NAD^+^@ysZIF-8 maintained structural integrity and good crystallinity after 10 days storage and after catalytic reaction post-storage. Furthermore, Pro@ZIF-8@ADH/NAD^+^@ysZIF-8 showed negligible enzyme leaching (Supplementary Fig. 40). To sum up, these results reveal that multi-shelled hollow ZIF-8 MOFs can spatially confine Pro and ADH/NAD^+^ in distinct compartments, allowing for an incompatible tandem biocatalytic reaction to be performed simultaneously in a single MOF nanoparticle, similar to natural cells.

### Position control of multi-enzyme cascade

**Fig. 3:**
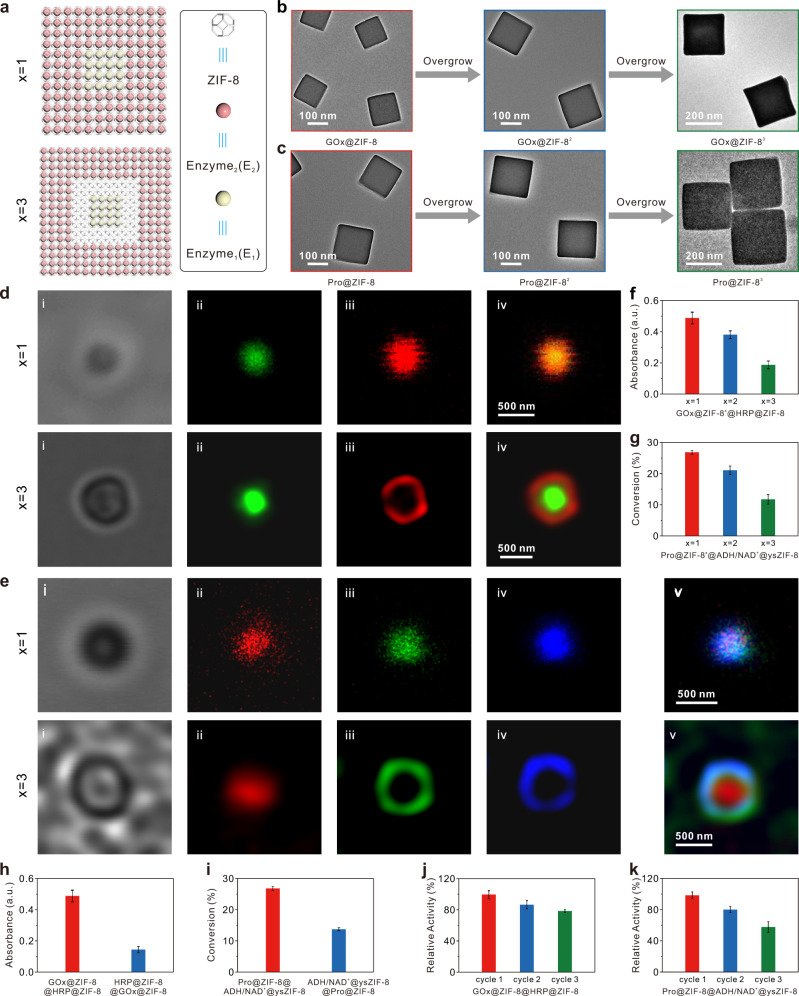
Position control of multi-enzyme cascade. **a** Schematic illustrating the interenzyme distance can be adjusted by tuning the epitaxial growth cycles of ZIF-8 (*x* = 1–3) in E_1_@ZIF-8^*x*^@E_2_@ZIF-8. TEM images of **b** GOx@ZIF-8^*x*^ (*x* = 1–3) and **c** Pro@ZIF-8^*x*^ (*x* = 1–3), respectively. **d** Confocal microscope images of GOx-FITC@ZIF-8^*x*^@HRP-RhB@ZIF-8: (i) bright-field, (ii) excitation at 488 nm and monitoring the fluorescence of the FITC-labeled GOx at 520 nm; (iii) excitation at 543 nm and monitoring the fluorescence of the RhB-labeled HRP at 620 nm; (iv) merged image. **e** Confocal microscope images of Pro-RhB@ZIF-8^*x*^@ADH-FITC/NAD^+^-coumarin@ysZIF-8: (i) bright-field, (ii) excitation at 543 nm and monitoring the fluorescence of the RhB-labeled Pro at 620 nm; (iii) excitation at 488 nm and monitoring the fluorescence of the FITC-labeled ADH at 520 nm; (iv) excitation at 405 nm and monitoring the fluorescence of the coumarin-labeled NAD^+^ at 480 nm; (v) merged image. The effect of interenzyme distance on the **f** GOx-HRP and **g** Pro-ADH/NAD^+^ cascade activities. The effect of enzyme positions on the **h** GOx-HRP and **i** Pro-ADH/NAD^+^ cascade activities. Catalytic recyclability of **j** GOx@ZIF-8@HRP@ZIF-8 and **k** Pro@ZIF-8@ADH/NAD^+^@ysZIF-8. In all GOx-HRP cascade experiments, the concentrations of GOx and HRP were 4.71 and 14.4 μg mL^−1^, respectively. In all Pro-ADH/NAD^+^ cascade experiments, the concentrations of Pro, ADH, and NAD^+^ used were 5.35, 19.3, and 17.1 μg mL^−1^. The error bars represent standard deviation based on three measurements.

Next, to show the ability to mimic biological systems, in which well-organized separation between enzymes shows a significant effect on cascade process, we adjusted the interenzyme distance by tuning the epitaxial growth cycles of ZIF-8^33,85^. As illustrated in Fig. 3a, E_1_@ZIF-8 was used as the core to perform the overgrowth of ZIF-8 shell, and the thus-formed E_1_@ZIF-8^2^ was then used as the new core for further overgrowth of E_2_@ZIF-8 to produce the multi-shelled (hollow) E_1_@ZIF-8^2^@E_2_@ZIF-8^86^. The ZIF-8 shells encapsulating enzymes can be spaced ~20 nm apart by solid ZIF-8 shell using GOx@ZIF-8^2^ or Pro@ZIF-8^2^ as the new core, and can be further increased to ~60 nm by increasing the epitaxial growth cycles of ZIF-8 (Fig. 3b, c and Supplementary Tables 5 and 6). We characterized and verified the spatial organization of multi-enzymes using fluorescence confocal microscopy. We labeled GOx-HRP with fluorescein isothiocyanate (FITC) and Rhodamine B (RhB). As displayed in Fig. 3d-*x*_1_, the excitation at 488 nm yielded the green fluorescence of the FITC-labeled GOx (ii); excitation at 543 nm yielded red fluorescence of the RhB-labeled HRP (iii); the merged image confirmed the successful confinement of GOx and HRP in multi-shelled ZIF-8 (iv). The colocalization revealed a slightly larger spot of RhB-labeled HRP than FITC-GOx, however, the limited spatial resolution of fluorescence confocal microscopy prevents more-precision localization. Only when the spacing between GOx- and HRP-loaded shells was further increased to ~60 nm (that is GOx@ZIF-8^3^@HRP@ZIF-8, Fig. 3d-*x*_3_), colocalization unraveled the spatial distribution of FITC-labeled GOx and RhB-labeled HRP. Similarly, we labeled Pro-ADH/NAD^+^ with RhB-FITC/coumarin. Figure 3e-*x*_1_ displays confocal microscope images that support the confinement of Pro, ADH, and NAD^+^ in the multi-shelled hollow ZIF-8, and when the spacing was further increased to ~60 nm, the spatial distribution of incompatible enzymes and cofactors can then be clearly resolved (Fig. 3e-*x*_3_).

Next, the effect of interenzyme distance on the cascade process was investigated. As displayed in Fig. 3f, increasing the spacing between GOx- and HRP-loaded shells led to monotonous decrease in the cascade activity (61.4% decrease for ~60 nm spacing). A similar distance-dependent trend was also observed in Pro-ADH/NAD^+^ cascade (56.3% decrease for ~60 nm spacing, Fig. 3g). This phenomenon was similar to the proximity effect observed in previous works^23,86^. In E_1_@ZIF-8^*x*^@E_2_@ZIF-8 (*x* > 1), the diffusion direction of intermediate products within the solid ZIF-8 shell lacks specificity, which, to some extent, resulting in relatively lower concentration of intermediate products in the vicinity of E_2_ sites and hence decreasing the overall catalytic efficiency. Note that the cascade activity decay along with the increased compartment spacing is particularly pronounced in GOx-HRP system, which is likely due to intermediate decay. Previous experimental and simulation studies^72^ have demonstrated that in the case of enzyme cascade with intermediate decay (for instance, self-decomposition of H_2_O_2_ in the GOx-catalase or GOx-HRP systems) in particular, co-encapsulating multiple enzymes in MOFs that mimics the substrate channeling could lead to faster reaction rate and higher production yield. This also accounts for slightly decreased activity observed in GOx@ZIF-8@HRP@ZIF-8 compared with GOx/HRP@ZIF-8 (Fig. 1k), which corresponds to positioning E_1_-loaded and E_2_-loaded shells from ideal proximity to near-ideal proximity. While in an incompatible-enzyme system, close positioning of E_1_-loaded and E_2_-loaded shells in multi-spatial-compartmental MOF nanoparticle that approaches near-ideal proximity, could lead to effective transportation of intermediate products between compartments, resulting in an efficient tandem biocatalytic reaction.

Subsequently, we investigated the effect of enzyme positions on cascade process. We performed the same set of experiments by changing the encapsulation sequences of enzymes, denoted as E_2_@ZIF-8@E_1_@ZIF-8. As illustrated in Fig. 3h, when HRP was positioned in the inner-shell and GOx was positioned in the outer-shell (i.e., HRP@ZIF-8@GOx@ZIF-8), the cascade activity was decreased by 70.7% compared to that in GOx@ZIF-8@HRP@ZIF-8. Similarly, nearly 49.8% decrease in cascade activity occurred when ADH/NAD^+^ was positioned in the inner-shell and Pro was positioned in the outer-shell (i.e., ADH/NAD^+^@ysZIF-8@Pro@ZIF-8, Fig. 3i). This finding is similar to previous work^87,88^, and could be attributed to the loss of intermediate products during mass transportation from E_1_ to E_2_, which is greater within E_1_@ZIF-8@E_1_@ZIF-8 than within E_2_@ZIF-8@E_1_@ZIF-8. When the first enzyme (E_1_: GOx or Pro) was positioned in the inner-shell compartment, intermediates produced from the first-step reaction (that is H_2_O_2_ or ethanol) diffuse along the microporous channel of ZIF-8 shell, without any loss to the bulk environment. On the contrary, when the enzyme position was reversed, because the diffusion direction lacks specificity, intermediates produced from the first-step reaction likely penetrates out into the bulk environment, not just into the intended compartment, resulting in reduced intermediate enrichment around the second enzyme active site (E_2_: HRP or ADH) and hence leading to decreased catalytic efficiency. Taken together, these results reveal that multi-shelled ZIF-8 MOFs enables facile control of multi-enzyme positions according to specific tandem reaction routes, in which close positioning of E_1_-loaded and E_2_-loaded shells along the inner-to-outer shells could facilitate tandem reaction with efficient mass transportation between spatially confined enzymes’ active sites.

Furthermore, the compartmentalization makes catalyst easily recoverable by centrifugation. As displayed in Fig. 3j, k, GOx@ZIF-8@HRP@ZIF-8 and Pro@ZIF-8@ADH/NAD^+^@ysZIF-8 retained 78.8% and 64.8% of the relative activities after 3 catalytic cycles. The loss of activity is likely due to good dispersibility in aqueous solution leading to partial recovery of multi-shelled MOF particles during centrifugation. The rational design and synthesis of micrometer-sized particles might potentially address this issue.

## Discussion

In conclusion, we have demonstrated that multi-shelled MOFs could be used as a hierarchical scaffold to spatially organize enzymes on the nanoscale to enhance catalytic efficiency. Compared with other strategies, using multi-shelled MOFs for designing multi-enzyme catalytic cascades provides several remarkable features: (i) The porous MOF scaffold provides a designated diffusion path for mass transfer between spatially confined enzymes’ active sites, promoting efficient operation of tandem biocatalytic reaction. (ii) The incompatible enzymes can be loaded independently in distinct domains of multi-shelled MOFs during epitaxial shell-by-shell overgrowth, providing spatial segregation for multi-step tandem reaction to occur simultaneously within single MOF nanoparticles in one pot. (iii) Multi-enzymes can be spatially confined with varied inter-enzyme distances by adjusting epitaxial overgrowth cycles, and with varied enzyme positions by adjusting encapsulation sequences; therefore, the tandem biocatalytic efficiency can be facilely regulated. Collectively, these features are anticipated to make multi-shelled MOFs as promising nanocarriers for multi-enzyme catalytic cascades in biotechnological and therapeutic applications^25,27,28^.

Future applications could be explored further in several directions. For instance, the established GOx@ZIF-8@HRP@ZIF-8 could serve as a glucose biosensor, wherein the MOF scaffold protects enzymes from decomposition within the cell yet confer cellular uptake. This could also be extended to combine more functional compartments for glucose-responsive drug delivery. Additionally, the established Pro@ZIF-8@ADH/NAD^+^@ysZIF-8 could be potentially applied for l-norvaline production with high yield and optical purity. Pro as well as aldehyde have been proven to be efficient enantioselective catalysts for amino acid ester racemization^89^. By using Pro-ADH/NAD^+^ cascade, Pro catalyzes the conversion of L-norvaline ethyl ester to L-norvaline and ethanol, followed by ADH catalyzing the oxidation of ethanol to acetaldehyde which also aids the racemization of l-norvaline ethyl ester. Compartmentalizing incompatible Pro and ADH/NAD^+^ in a single MOF nanoparticle would potentially not only allow efficient racemization reaction in one pot but also improve the stability to harsh conditions (e.g., high temperature, organic solvents). Moreover, independent loading via epitaxial shell-by-shell overgrowth will make customizing multi-enzyme cascades more feasible. For instance, by confining incompatible GOx and MNP, we could develop a glucose-powered therapy for eliminating target cancer cells that do not require the use of toxic cancer drugs^90^. Also note that these systems could be combined with magnetically responsive component, thus facilitating separation and improving the recyclability of the biocatalysts on demand. We believe that this strategy is likely to be broadly beneficial to the fields of biochemical process engineering and disease therapeutics.

## Methods

### Materials

Glucose oxidase (GOx), peroxidase from horseradish (HRP), alcohol dehydrogenase (ADH), nicotinamide adenine dinucleotide hydrate (NAD^+^), trypsin, d-glucose, and alkaline protease were purchased from Sangon Biotech Co. Ltd. (Shanghai, China). Cobalt (II) nitrate hexahydrate (99.998%), zinc nitrate hexahydrate (99.998%), Rhodamine B, o-phenylenediamine (OPD), N,N-dimethyl-Formamide (DMF), aldehyde and 2-methylimidazole, Rhodamine B isothiocyanate, and 7-hydroxycoumarin-3-carboxylic acid *N*-succinimidyl ester were purchased from Sigma-Aldrich were purchased from Sigma-Aldrich. l-Norvaline ethyl ester hydrochloride, fluorescein isothiocyanate (FITC), coumarin, and cetyltrimethylammonium bromide (CTAB) were purchased from Aladdin. All the other reagents were purchased from Sinopharm Chemical Reagent, Co. Ltd. and used without further purification. Ultrapure water (≥18.2 MΩ cm) was used throughout the study.

### Characterizations

TEM images were obtained on JEOL JEM2010 operated at 200 kV. TGA was conducted on a METTLER TOLEDO’s SDTA851e simultaneous thermal analyzer, collecting the thermogravimetric change data from 25 °C to 800 °C in a continuous flow of nitrogen for ~5 mg samples. Powder XRD patterns were recorded using a Rigaku Corporation’s Ultima IV X-ray diffractometer. FTIR spectra were collected using the iS50 Fourier transform infrared spectrometer from Nicolet. Particle size distributions were obtained using NANO ZS360 NANO particle size. STEM and EDX mapping experiments were performed on a FEI Probe Cs-corrected Titan operating at 200 kV. Confocal microscopy images were recorded using a Leica TCS SP8 STED 3X confocal laser scanning microscope to determine the presence and spatial location of the fluorophore-tagged enzymes in the multi-shelled (hollow) ZIF-8 MOFs. Ultraviolet-visible absorption spectra were collected using Agilent Cary 60 spectrophotometer. The nitrogen gas adsorption–desorption was measured at 77 K on a Quantachrome Autosorb-iQ-MP volumetric gas adsorption analyzer. Gas chromatography (GC) was conducted on Shimadzu’s GC-2010 pro.

### Synthesis of ZIF-8

1.75 mL of 2-methylimidazole (790 mM) containing 550 µM CTAB was stirred at 500 rpm for 5 min, then 0.25 mL Zn(NO_3_)_2_·6H_2_O aqueous solution (97.5 mM) was added. After stirring for 5 min, the mixture was left undisturbed for 3 hr at room temperature. Finally, the prepared ZIF-8 was collected by centrifugation at 3500×*g* for 10 min, and dispersed into 1 mL water. Note that surfactant CTAB was utilized as a capping agent to control the size and shape of ZIF-8 in an aqueous solution based on the previous reports^91^.

### Synthesis of GOx@ZIF-8 and HRP@ZIF-8

A certain amount of GOx (or HRP) was added into 1.75 mL of 2-methylimidazole (790 mM) containing 550 µM CTAB under stirring at 500 rpm, followed by the addition of 0.25 mL Zn(NO_3_)_2_·6H_2_O aqueous solution (97.5 mM). After stirring for 5 min, the mixture was left undisturbed for 3 h at room temperature. Finally, the prepared GOx@ZIF-8 (or HRP@ZIF-8) was collected by centrifugation at 3500 g for 10 min, washed with water two times, and dispersed into 1 mL water.

### Synthesis of GOx@ZIF-8@HRP@ZIF-8 and HRP@ZIF-8@GOx@ZIF-8

0.2 mL as-synthesized GOx@ZIF-8 (or HRP@ZIF-8) was added into 1.75 mL of 2-methylimidazole (790 mM) containing 550 µM CTAB under stirring at 500 rpm, followed by addition of HRP and the mixture was stirred for another 1 min at 500 rpm, then 0.25 mL Zn(NO_3_)_2_·6H_2_O aqueous solution (97.5 mM) was added. After stirring for 5 min, the mixture was left undisturbed for 1 h at room temperature. Finally, the prepared GOx@ZIF-8@HRP@ZIF-8 (or HRP@ZIF-8@GOx@ZIF-8) was collected by centrifugation at 3500×*g* for 10 min, washed with water two times, and dispersed into 1 mL water.

### Synthesis of GOx@ZIF-8^2^@HRP@ZIF-8

1 mL as-synthesized GOx@ZIF-8 was added into 1.75 mL of 2-methylimidazole (790 mM) containing 550 µM CTAB under stirring at 500 rpm, followed by addition of 0.25 mL Zn(NO_3_)_2_·6H_2_O aqueous solution (97.5 mM). After stirring for 5 min, the mixture was left undisturbed for 1 h at room temperature. Finally, the prepared GOx@ZIF-8^2^ was collected by centrifugation at 3500 × *g* for 10 min and dispersed into 1 mL water. GOx@ZIF-8^2^@HRP@ZIF-8 was then synthesized following the same protocol of GOx@ZIF-8@HRP@ZIF-8 except using GOx@ZIF-8^2^ as the core.

### Synthesis of Pro@ZIF-8

A certain amount of Pro was added into 1.75 mL of 2-methylimidazole (790 mM) containing 550 µM CTAB under stirring at 500 rpm, followed by the addition of 0.25 mL Zn(NO_3_)_2_·6H_2_O aqueous solution (97.5 mM). After stirring for 5 min, the mixture was left undisturbed for 3 h at room temperature. Finally, the prepared Pro@ZIF-8 was collected by centrifugation at 3500 × *g* for 10 min, washed by water for two times, and dispersed into 1 mL water.

### Synthesis of Pro@ZIF-8@ADH/NAD^+^@ysZIF-8

0.2 mL as-synthesized Pro@ZIF-8 was added into 1.75 mL of 2-methylimidazole (790 mM) containing 550 µM CTAB under stirring at 500 rpm, followed by addition of ADH and the mixture was stirred for another 1 min at 500 rpm, then add 0.25 mL Co(NO_3_)_2_·6H_2_O aqueous solution (97.5 mM). After stirring for 5 min, the mixture was left undisturbed for 1 h at room temperature. The prepared Pro@ZIF-8@ADH/NAD^+^@ZIF-67 particles were collected by centrifugation at 3500 × *g* for 10 mins and washed with water two times. The synthesized Pro@ZIF-8@ADH/NAD^+^@ZIF-67 was dispersed in 2.5 mL methanol containing 30 mM 2-methylimidazole, after shaking 5 s, then 2.5 mL 30 mM Zn(NO_3_)_2_·6H_2_O methanol solution was injected. The mixture was left undisturbed at room temperature for 1 h. Then, the prepared Pro@ZIF-8@ADH/NAD^+^@ZIF-67@ZIF-8 were collected by centrifugation at 3500 × *g* for 10 mins, and dispersed in volume ratio 1:1 water and methanol solution to dissociate the ZIF-67 for the formation of Pro@ZIF-8@ADH/NAD^+^@ysZIF-8. The ADH/NAD^+^@ysZIF-8@Pro@ZIF-8 was similarly prepared by reversing the encapsulation sequence.

### Synthesis of Pro@ZIF-8^2^ and Pro@ZIF-8^2^@ADH/NAD^+^@ysZIF-8

The process for the preparation of Pro@ZIF-8^2^@ADH/NAD^+^@ysZIF-8 was the same as the synthesis of GOx@ZIF-8^2^@HRP@ZIF-8 except using Pro@ZIF-8^2^ as the core.

### Preparation of dye-labeled enzymes

Enzymes were labeled using amine-reactive fluorophores following previously reported protocol.^41,56^ 20-fold of FITC, Rhodamine B isothiocyanate, or 7-hydroxycoumarin-3-carboxylic acid *N*-succinimidyl ester was mixed with GOx/ADH, Pro/HRP, or NAD^+^ in 0.5 M carbonate buffer (pH = 8.3), respectively. After 6 h, the excess dyes were removed with 3-kD cutoff Amicon filters. For detailed mechanism, please see Supplementary Fig. 41.

### Encapsulation efficiencies of enzymes in ZIF-8

The encapsulation efficiencies of compatible enzymes in ZIF-8 were evaluated by labeling GOx with FITC and HRP with Rhodamine B, respectively. Specifically, after encapsulation of dye-labeled enzymes into ZIF-8, fluorescence spectra of supernatant were recorded, the enzyme concentration in the supernatant was then determined using the corresponding calibration curves of dyes (see Supplementary Fig. 5). The encapsulation efficiencies were then determined as the ratio of enzymes content embedded in the enzyme@ZIF-8 composites to the initial amount of enzyme, which was calculated based on the following equation:

1 $${{{{{\rm{encapsulation}}}}}}\; {{{{{\rm{efficiency}}}}}}( \% )=\frac{m-{C}_{1}{V}_{1}}{m}$$

Where *m* (mg) is the initially added enzyme amount, *C*_1_ (mg/mL) is the enzyme concentration in the supernatant, and *V*_1_ (mL) is the volume of supernatant. Likewise, Pro, ADH, and NAD^+^ were labeled with FITC, Rhodamine B, and coumarin, respectively. The encapsulation efficiencies of noncompatible enzymes were evaluated by fluorescent spectrophotometry using the respective calibration curves (see Supplementary Fig. 26). The encapsulation efficiencies of enzymes in different systems are listed in Supplementary Table 2. Given that encapsulation efficiencies were different in different systems, the initial amounts of enzymes added in the synthetic protocol were varied (listed in Supplementary Table 7) to ensure that the final enzyme concentrations were consistent across different control experiments.

### Operation of enzyme cascade in GOx@ZIF-8@HRP@ZIF-8

500 µL glucose solution (80 µM) and 500 µL OPD solution (10 mM) were added to GOx@ZIF-8@HRP@ZIF-8 composite, followed by incubation at room temperature for 11 min. Then, the reaction solution was centrifuged at 10000 rpm for 3 min to remove the GOx@ZIF-8@HRP@ZIF-8 composite and the absorbance at 417 nm was recorded on Agilent Cary 60 ultraviolet-visible spectrophotometer. In all control experiments, including free GOx & HRP, GOx@ZIF-8 & HRP@ZIF-8, GOx@ZIF-8^*x*^@HRP@ZIF-8, HRP@ZIF-8@GOx@ZIF-8 systems, and GOx@ZIF-8@HRP@ZIF-8, the same amount of enzymes (the concentrations of GOx and HRP were 4.71 and 14.4 μg mL^−1^, respectively) were used on the basis of the fluorescence quantification results.

### Trypsin digestion

The suspension of GOx@ZIF-8@HRP@ZIF-8 or free GOx & HRP was incubated with 50 µg/mL of trypsin at 37 °C for 24 h. The residual was then used for cascadic reaction following the standard protocol.

### Operation of enzyme cascade in Pro@ZIF-8@ADH/NAD^+^@ysZIF-8

The typical cofactor-enzyme cascade assay was conducted in 1 mL of l-norvaline ethyl ester hydrochloride solution (50 mM) and as-synthesized Pro@ZIF-8@ADH/NAD^+^@ysZIF-8. The conversion rate of the cofactor-enzyme cascade reaction was monitored by the detection of acetaldehyde using SHIMADZU’s GC-2010 pro gas chromatography. In all control experiments, including free Pro & ADH/NAD^+^, Pro@ZIF-8 & ADH/NAD^+^@ysZIF-8, Pro@ZIF-8^*x*^@ADH/NAD^+^@ysZIF-8, and ADH/NAD^+^@ysZIF-8@Pro@ZIF-8 systems, the same amount of enzymes (the concentrations of Pro, ADH, and NAD^+^ were 5.35, 19.3, and 17.1 μg mL^−1^, respectively) were used on the basis of the fluorescence quantification results.

### Molecular size calculations

The molecular sizes of glucose and l-norvaline ethyl ester hydrochloride were estimated by using VMD software package. The longest interatomic distances for each molecule are taken as the effective molecular sizes (Supplementary Fig. 12).

### Nano-FTIR

Nanoscale infrared spectroscopy and imaging were conducted with a commercial s-SNOM/nano-FTIR setup (NeaSNOM; Neaspec GmbH, Germany), as illustrated in Fig. 1e. For nano-FTIR spectra, a broadband infrared laser continuum with an average output power of ~600 μW was used. The AFM was operated in ~250-kHz tapping mode with metallic tips (Arrow PtIr; NanoWorld AG). All nano-FTIR spectra were recorded with 10 cm^−1^ spectral resolution and a 10 nm tapping amplitude. For imaging, a wavelength-tunable quantum cascade laser (QCL) was used with an average output power of ~3 mW and a tapping amplitude of ~10 nm.

## Supplementary information

Supplementary Information

## Data Availability

All the data that support the findings of this study are available within the paper and its Supplementary Information files, and from the corresponding authors upon request. Source data for Figs. 1–3 and the Supplementary Figs. are provided as a Source Data file. Source data are provided with this paper.

## Source data

Source Data
