# Supplementary material for: Hierarchically encapsulating enzymes with multi-shelled metal-organic frameworks for tandem biocatalytic reactions
Source: Nat Commun. 2022 Jan 13;13:305. doi: 10.1038/s41467-022-27983-9 (PMC8758787; doi:10.1038/s41467-022-27983-9)
Supplement: Supplementary file 1 — Supplementary Information [file 41467_2022_27983_MOESM1_ESM.pdf]

Hierarchically encapsulating enzymes with multi-shelled metal-organic frameworks for tandem biocatalytic reactions

Tiantian Man[1,2], Caixia Xu[1], Xiao-Yuan Liu[3], Dan Li[1], Chia-Kuang Tsung[4], Hao Pei[1], Ying Wan[2] & Li Li[1,*]

[1]Shanghai Key Laboratory of Green Chemistry and Chemical Processes, School of Chemistry and Molecular Engineering, East China Normal University, 500 Dongchuan Road, Shanghai 200241, P. R. China

[2]School of Mechanical Engineering, Nanjing University of Science and Technology, Nanjing 210094, P. R. China

[3]Hoffmann Institute of Advanced Materials, Shenzhen Polytechnic, 7098 Liuxian Boulevard, Nanshan District, Shenzhen 518055, P. R. China

[4]Department of Chemistry, Merkert Chemistry Center, Boston College, 2609 Beacon Street, Chestnut Hill, Massachusetts 02467, United States

*Corresponding author. Email: lli@chem.ecnu.edu.cn

The materials include content:

Supplementary Figs 1 to 41

Supplementary Tables 1 to 7

Supplementary References

Supplementary Figures

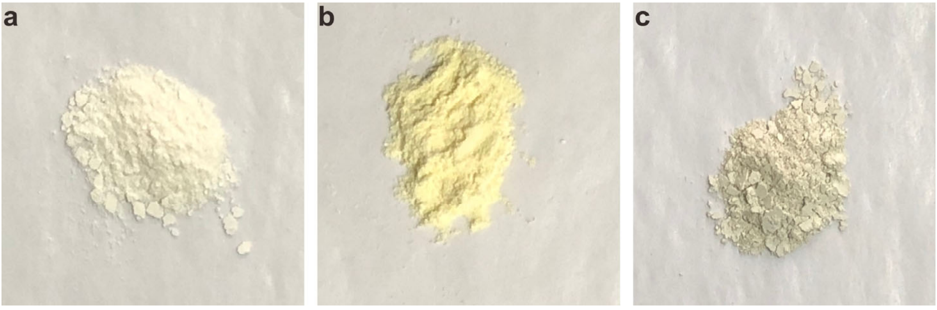

Supplementary Fig. 1 Photographs of as-synthesized a) ZIF-8, b) GOx@ZIF-8, and c) GOx@ZIF-8@HRP@ZIF-8.

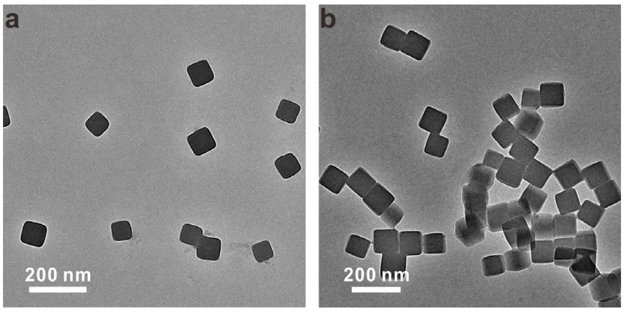

Supplementary Fig. 2 TEM images of as-synthesized a) ZIF-8 and b) GOx@ZIF-8.

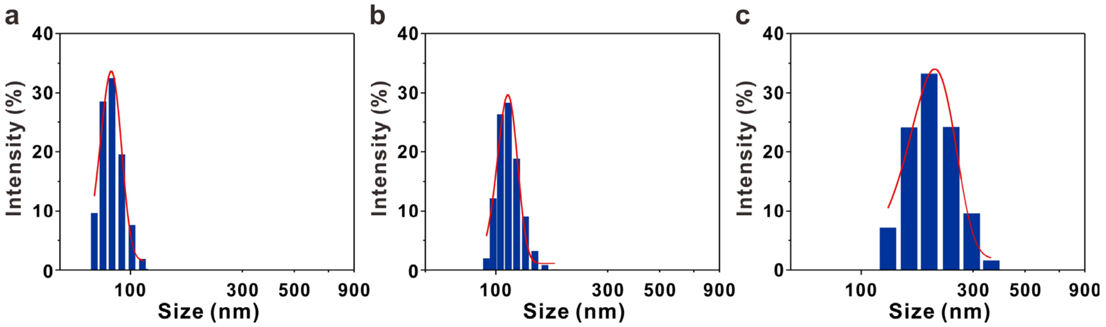

Supplementary Fig. 3 Dynamic light scattering size measurements of as-synthesized a) ZIF-8 (91±16 nm), b) GOx@ZIF-8 (143±21 nm), and c) GOx@ZIF-8@HRP@ZIF-8 (205±93 nm).

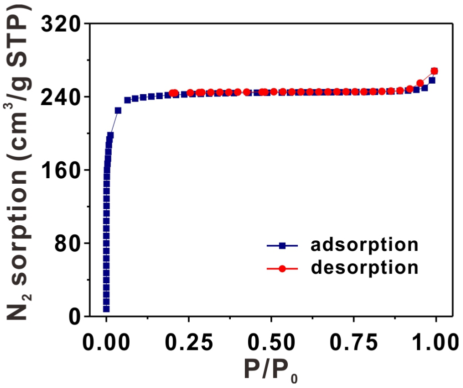

Supplementary Fig. 4 Nitrogen adsorption isotherm and desorption isotherm of as-synthesized GOx@ZIF-8@HRP@ZIF-8.

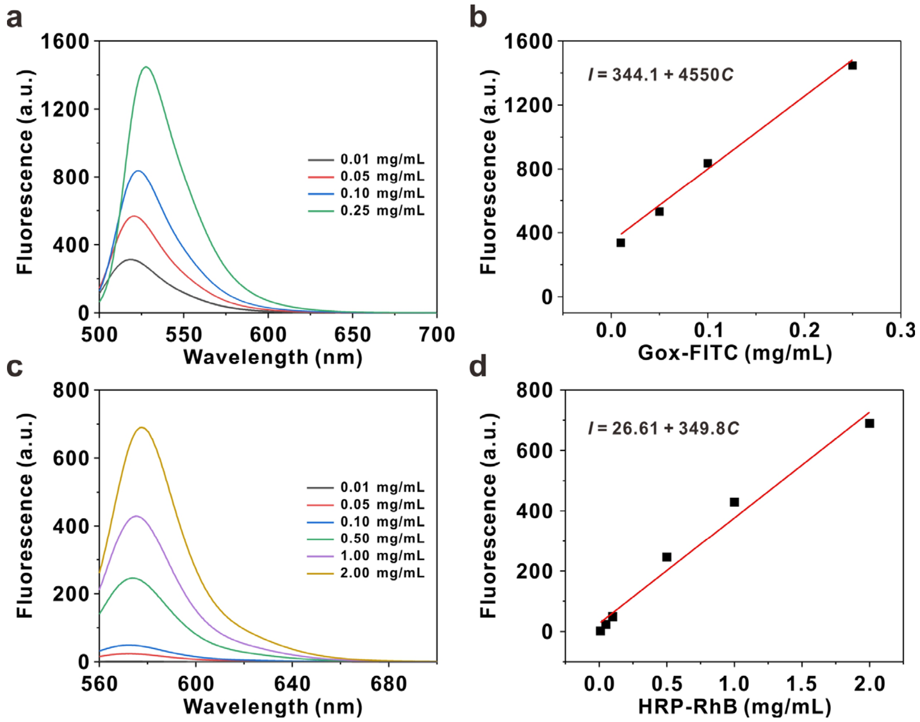

Supplementary Fig. 5 Fluorescence spectra of different concentrations of the a) GOx-FITC and c) HRP-RhB, respectively. Calibration curves of b) GOx-FITC and d) HRP-RhB.

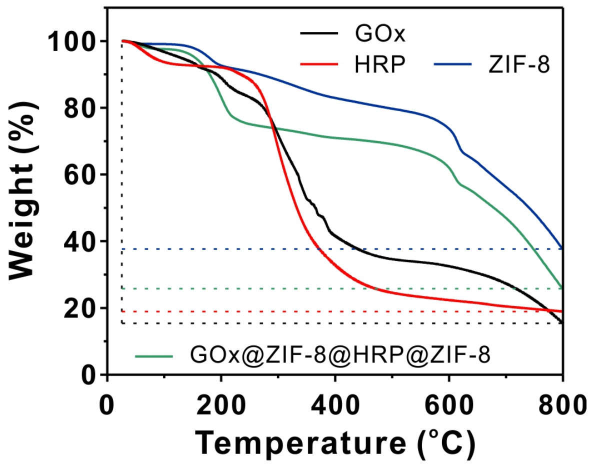

Supplementary Fig. 6 TGA curves of GOx (black), HRP (red), ZIF-8 (blue) and GOx@ZIF-8@HRP@ZIF-8 (green).

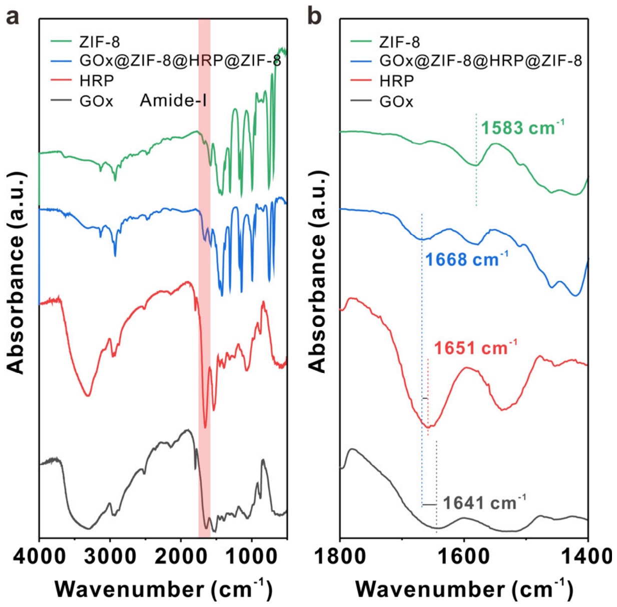

Supplementary Fig. 7 a) FTIR spectra and b) magnified FTIR spectra of as-synthesized ZIF-8, GOx@ZIF-8@HRP@ZIF-8, HRP, and GOx.

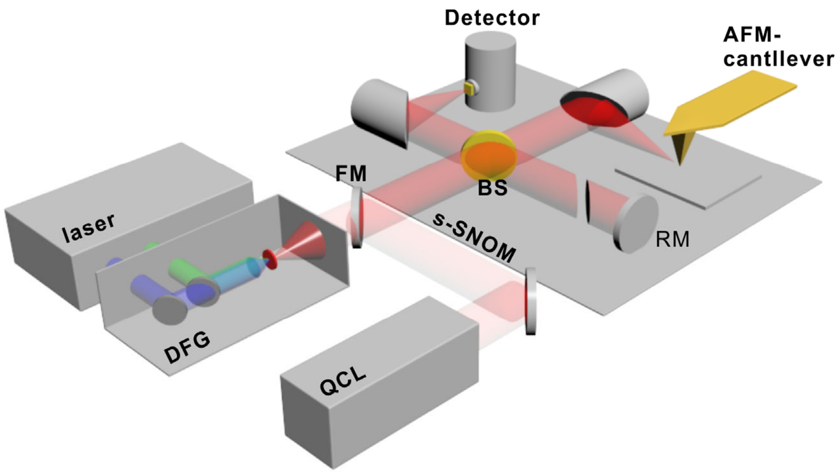

Supplementary Fig. 8 A commercial nano-FTIR set-up (Neaspec GmbH) employing a mid-infrared continuum source (A difference frequency generator (DFG) unit superimposes both beams in a nonlinear crystal, which subsequentially emits a mid-infrared continuum beam) or a tunable single line laser (quantum cascade laser, QCL) for tip illumination, respectively. The illuminating infrared beam (either from a broadband mid-infrared continuum source for point-scanning and line-scanning or from a tunable QCL for imaging) is focused onto the probe tip. The illumination can be chosen with a flip mirror (FM). The probe is typically a metallic atomic force microscope (AFM) tip, which can act as an antenna and concentrates the incident light at the tip apex. The light backscattered from the tip is analysed with an asymmetric Michelson interferometer comprising a beam splitter (BS, uncoated ZnSe), a reference mirror (RM), and a detector. The interferometric detection provides the sensitivity to measure the sample's permittivity with an ultrahigh resolution dependent only on the size of the tip apex (typically down to 20 nm). Demodulating the detector signal at higher harmonics of the tip oscillation frequency (nΩ) extracts the complex-valued near-field interaction, whose imaginary part defines the local nanoFTIR absorption. Earlier experimental and theoretical studies[1-3] indicate that nano-FTIR near-field absorption spectra match well with conventional far-field absorption spectra, including spectral line positions, line widths, and line shapes. On this basis, nano-FTIR spectroscopy could allow for direct chemical recognition of materials in ultrasmall quantities and at ultrahigh spatial resolution by consulting standard FTIR databases.

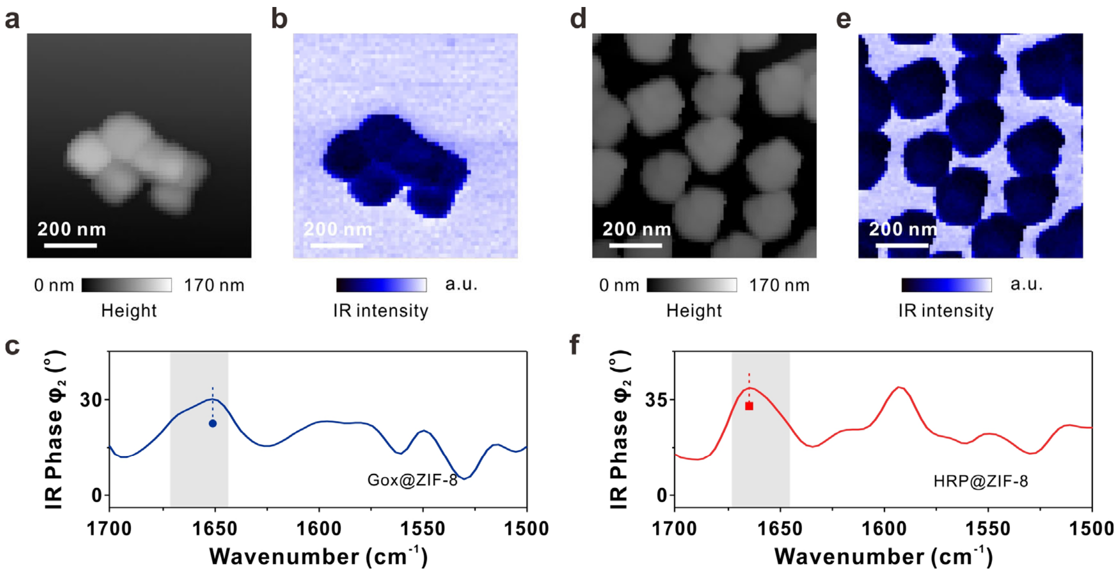

Supplementary Fig. 9 Simultaneously recorded a, d) AFM topography images and b, e) IR broadband images of GOx@ZIF-8 and HRP@ZIF-8 particles, respectively. Representative point nano-FTIR spectra of c) GOx@ZIF-8 and f) HRP@ZIF-8 particles.

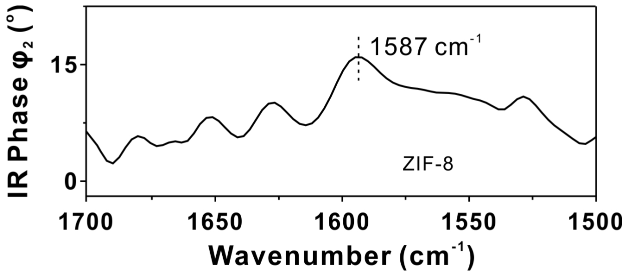

Supplementary Fig. 10 Representative point nano-FTIR spectra of ZIF-8 particles.

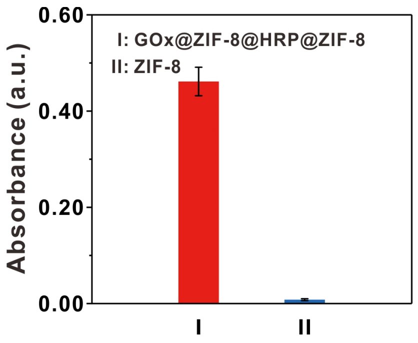

Supplementary Fig. 11 Catalytic efficiencies in GOx@ZIF-8@HRP@ZIF-8 and ZIF-8.

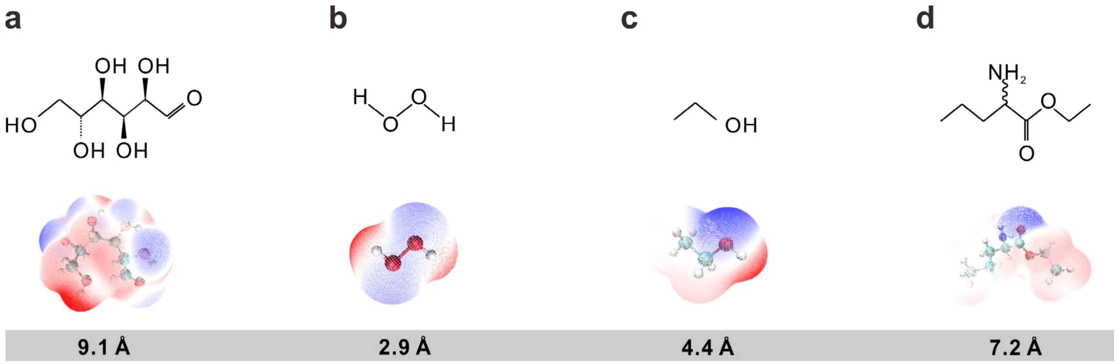

Supplementary Fig. 12 Molecular sizes of a) glucose, b) H_2O_2, c) EtOH, and d) L-norvaline ethyl ester.

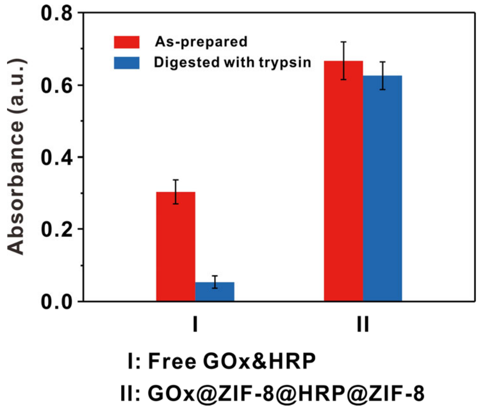

Supplementary Fig. 13 Comparison of cascade activities before (red) and after digestion with trypsin (blue). For free GOx&HRP: concentrations of GOx and HRP were 15.0 and 45.9 μg mL^{-1}. For GOx@ZIF-8@HRP@ZIF-8: concentrations of GOx and HRP were 6.6 and 20.2 μg mL^{-1}.

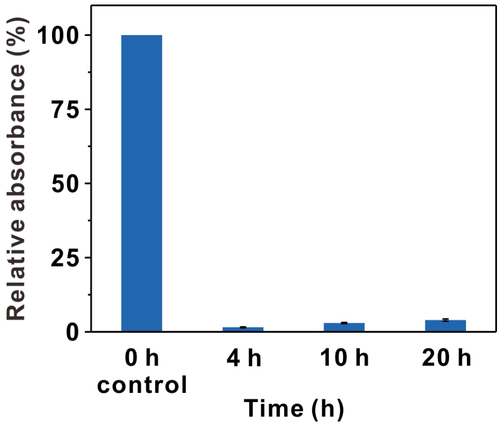

Supplementary Fig. 14 Leaching test of GOx@ZIF-8@HRP@ZIF-8 at 4 h, 10 h and 20 h. The amount of leaching enzymes was determined by Bradford assay. The 100% standard solution at 0 minute was composed of 4.71 μg mL^{-1} GOx and 14.4 μg mL^{-1} HRP in water.

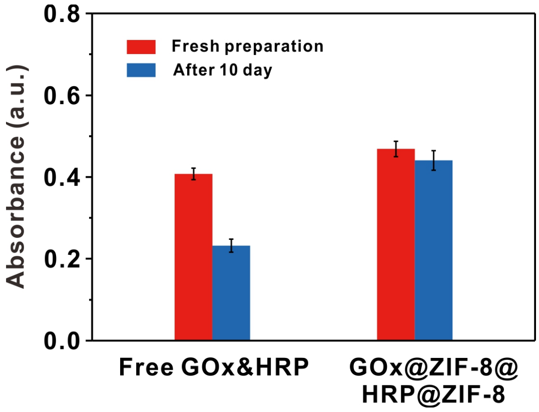

Supplementary Fig. 15 Comparison of long-term stability. Cascade activities of the free GOx&HRP and GOx@ZIF-8@HRP@ZIF-8 immediately after fresh preparation (red) and after storage at room temperature for a time-interval of 10 day (blue). For free GOx&HRP: concentrations of GOx and HRP were 25.0 and 76.4 μg mL^{-1}. For GOx@ZIF-8@HRP@ZIF-8: concentrations of GOx and HRP were 4.71 and 14.4 μg mL^{-1}.

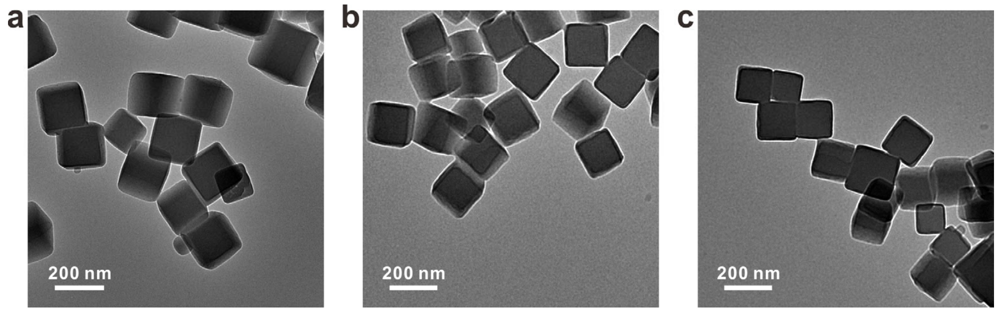

Supplementary Fig. 16 TEM images of GOx@ZIF-8@HRP@ZIF-8 a) immediately after preparation, b) after 10 day of storage at 4 °C in pure water, and c) after catalytic reaction post-storage.

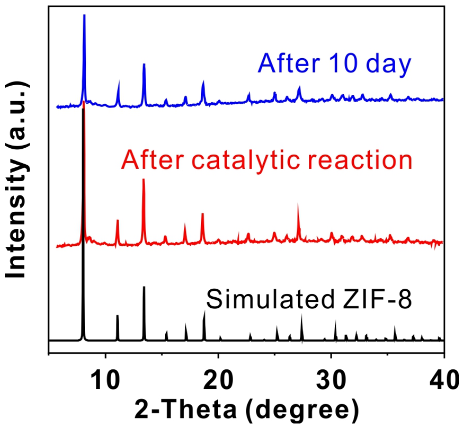

Supplementary Fig. 17 XRD pattern simulated from CIF file of ZIF-8 (black), XRD patterns of the GOx@ZIF-8@HRP@ZIF-8 after storage at room temperature for a time-interval of 10 day (blue) and after catalytic reaction post-storage (red).

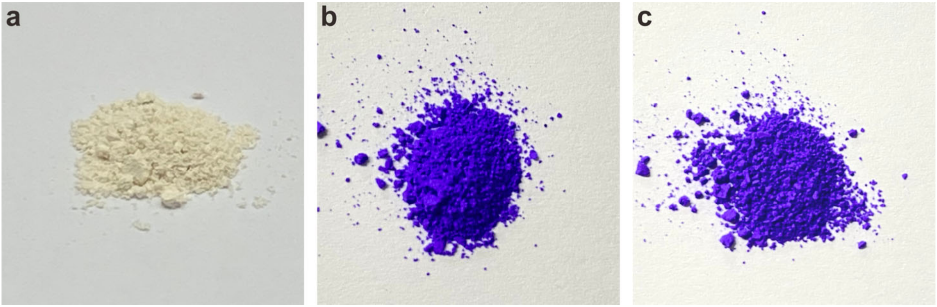

Supplementary Fig. 18 Photographs of as-synthesized a) Pro@ZIF-8, b) Pro@ZIF-8@ADH/NAD$^+$@ZIF-67@ZIF-8, and c) Pro@ZIF-8@ADH/NAD$^+$@ysZIF-8.

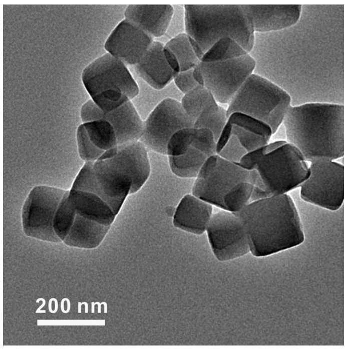

Supplementary Fig. 19 TEM image of as-synthesized Pro@ZIF-8.

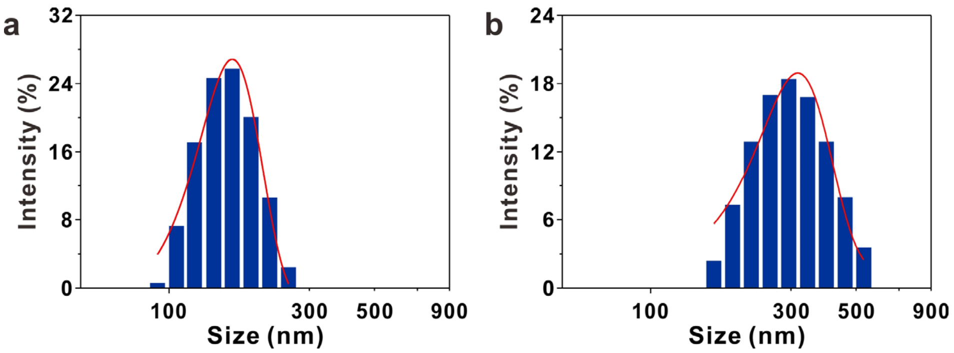

Supplementary Fig. 20 Dynamic light scattering size measurements of as-synthesized Pro@ZIF-8 (164±37 nm) and b) Pro@ZIF-8@ADH/NAD$^+$@ysZIF-8 (306±90 nm).

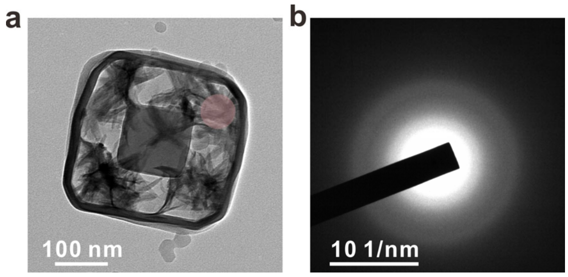

Supplementary Fig. 21 a) TEM image and b) selected area electron diffraction (SAED) diffraction pattern of as-synthesized Pro@ZIF-8@ADH/NAD$^+$@ysZIF-8 conforming the amorphous state of cobalt hydroxides.

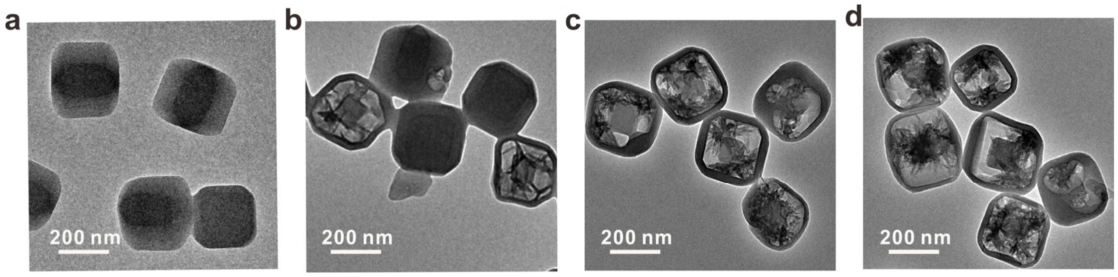

Supplementary Fig. 22 TEM images of Pro@ZIF-8@ADH/NAD$^+$@ZIF-67@ZIF-8 with different etching time of a) 0 day, b) 1 day, c) 3 day, and d) 7 day.

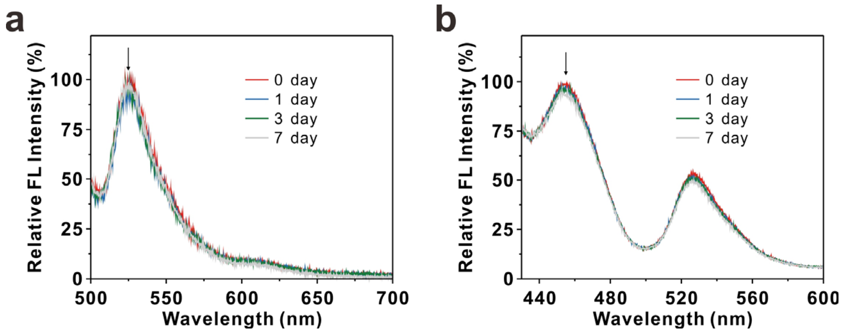

Supplementary Fig. 23 Fluorescence spectra of Pro@ZIF-8@ADH-FITC/NAD$^+$-coumarin@ZIF-67@ZIF-8 composites a) excited at 488 nm and monitoring the fluorescence of FITC-labelled ADH; b) excited at 405 nm and monitoring the fluorescence of coumarin-labelled NAD$^+$ with different etching time of 0 h, 1 day, 3 day, and 7 day. Note that the fluorescence of enzyme-MOF composites was recorded during the hollowing process since we could not observe any fluorescent signal from the supernatant.

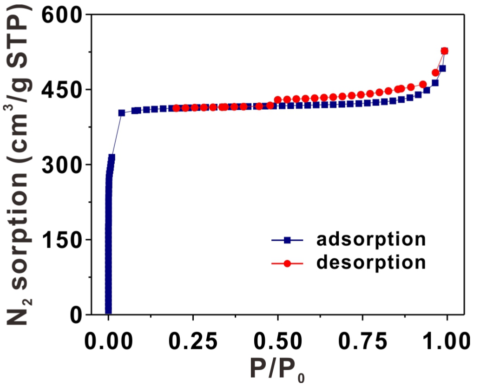

Supplementary Fig. 24 Nitrogen adsorption isotherm and desorption isotherm of as-synthesized Pro@ZIF-8@ADH/NAD$^+$@ysZIF-8.

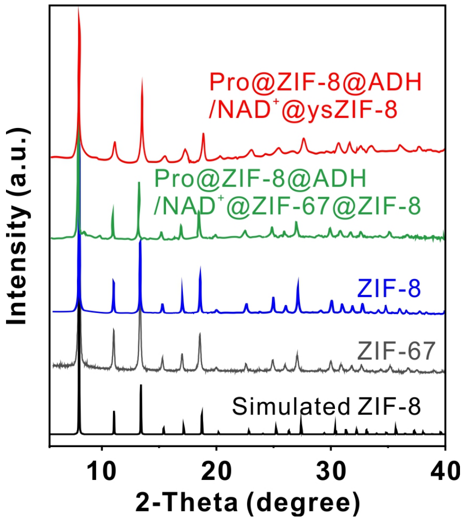

Supplementary Fig. 25 XRD pattern simulated from CIF file of ZIF-8 (black), XRD patterns of synthesized ZIF-67 (gray) and ZIF-8 (blue), Pro@ZIF-8@ADH/NAD$^+$@ZIF-67@ZIF-8 (green), and Pro@ZIF-8@ADH/NAD$^+$@ysZIF-8 (red).

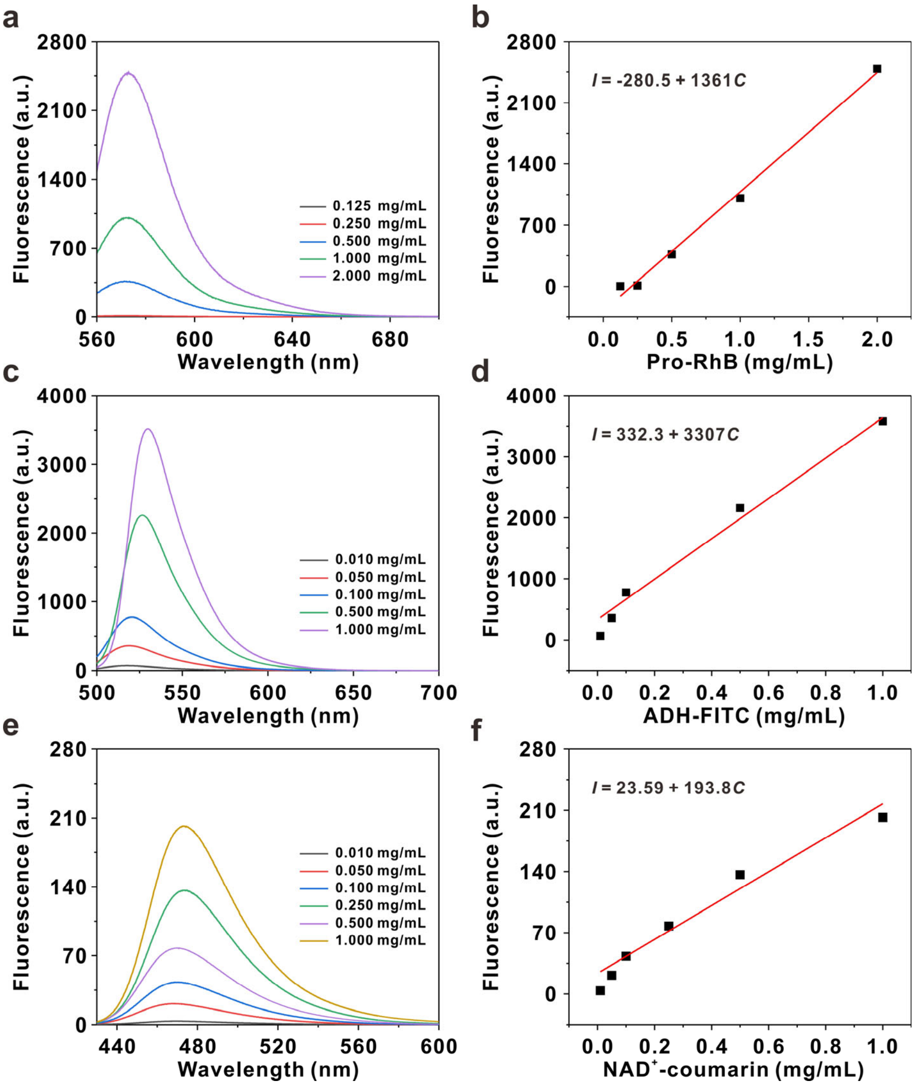

Supplementary Fig. 26 Fluorescence spectra of different concentrations of the a) Pro-RhB, c) ADH-FITC, and e) NAD$^+$-coumarin, respectively. Calibration curves of b) Pro-RhB, d) ADH-FITC, and f) NAD$^+$-coumarin.

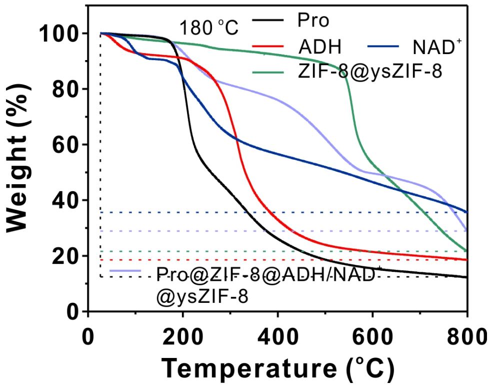

Supplementary Fig. 27 TGA curves of Pro (black), ADH (red), NAD$^+$ (blue), ZIF-8@ysZIF-8 (green) and Pro@ZIF-8@ADH/NAD$^+$@ysZIF-8 (light blue).

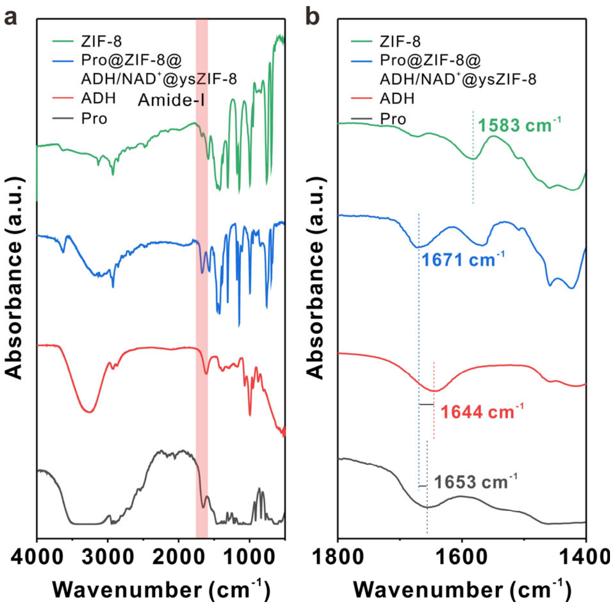

Supplementary Fig. 28 a) FTIR spectra and b) magnified FTIR spectra of as-synthesized ZIF-8, Pro@ZIF-8@ADH/NAD$^+$@ysZIF-8, Pro, and ADH.

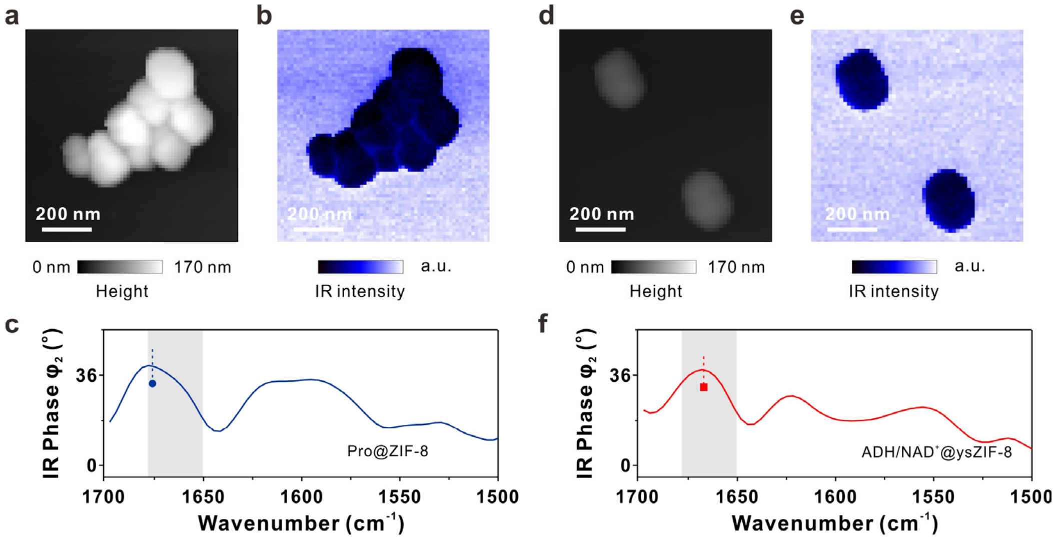

Supplementary Fig. 29 Simultaneously recorded a, d) AFM topography images and b, e) IR broadband images of Pro@ZIF-8 and ADH/NAD$^+$@ysZIF-8 particles, respectively. Representative point nano-FTIR spectra of c) Pro@ZIF-8 and f) ADH/NAD$^+$@ysZIF-8 particles.

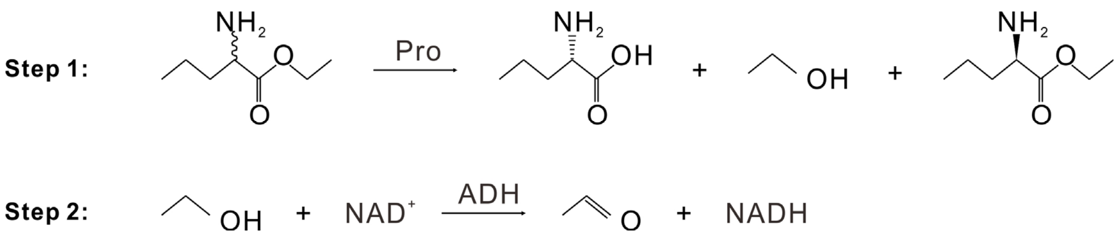

Supplementary Fig. 30 Tandem biocatalytic reaction driven by incompatible enzymes and cofactor (Pro and ADH/NAD$^+$).

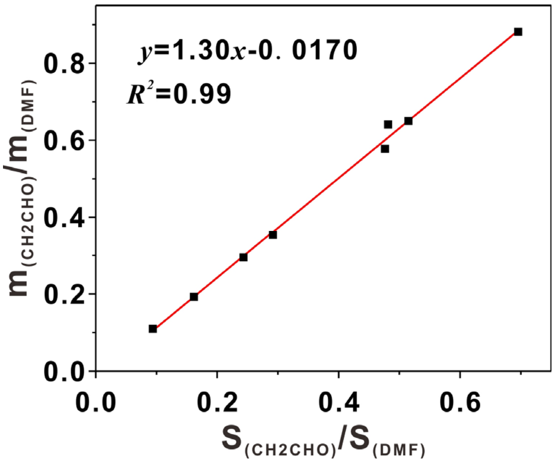

Supplementary Fig. 31 Standard curve for determination of acetaldehyde content using gas chromatography.

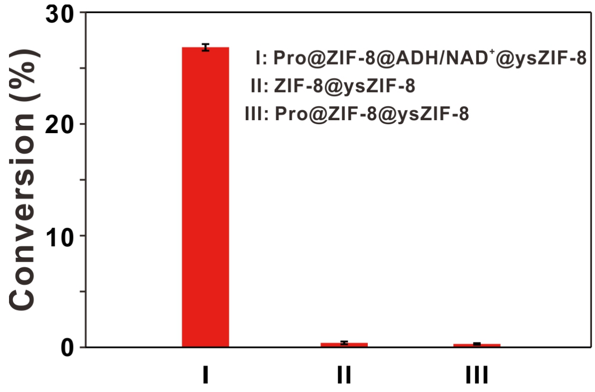

Supplementary Fig. 32 Catalytic efficiencies in Pro@ZIF-8@ADH/NAD$^+$@ysZIF-8, ZIF-8@ysZIF-8 and Pro@ZIF-8@ysZIF-8. The concentrations of Pro, ADH and NAD$^+$ used were 5.35, 19.3 and 17.1 μg mL^{-1}.

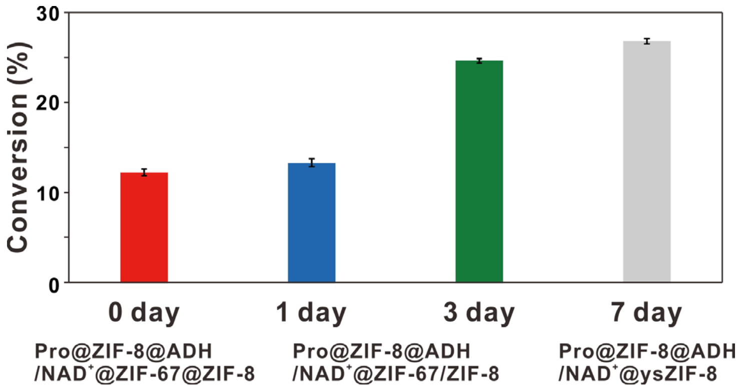

Supplementary Fig. 33 Catalytic efficiencies of Pro@ZIF-8@ADH/NAD$^+$@ZIF-67@ZIF-8 with different etching time of 0 day, 1 day, 3 day, 7 day. The concentrations of Pro, ADH and NAD$^+$ used were 5.35, 19.3 and 17.1 μg mL^{-1}.

NAD^{+}-dependent ADH uses the interconversion of NAD^{+}/NADH redox couple to catalyze the oxidation of alcohol to aldehyde. ADH exists as a dimer (that is, composed of two polypeptides), with each monomer containing a catalytic domain (that is catalytic zinc, which holds hydroxyl group on alcohol) and a coenzyme binding domain with a large cleft between the two. The active site is at the bottom of the cleft. According to previous studies[4-6], the mechanism of NAD^{+}-dependent ADH enzyme for the oxidation of alcohol to aldehyde is described as follows (summarized in Supplementary Fig. 34):

First, the ADH structure is initially open to facilitate access to the active site for NAD^{+} binding. When NAD^{+} and alcohol bind, ADH undergoes a global conformational change, which involves a rotation of the catalytic zinc domain relative to the NAD^{+} binding domain. This process closes up and isolates the active site from solvent, creating a hydrophobic environment for the productive holoenzyme complex (that is, E → E-NAD^{+} → E*-NAD^{+} → E*-NAD^{+}-RCH$_2$OH).

Second, the resulting E-NAD^{+}-RCH$_2$OH complex is poised for hydrogen transfer, involving alcohol to deprotonate, and transfer the proton via Ser-48 and His-51 (His-51 contacts solvent water on the protein surface) to solvent. During the proton relay, the reduced nicotinamide ring may become puckered. This process leads to the formation of E-NADH-RCHO.

Third, NADH and aldehyde dissociate from the abortive E-NADH-RCHO complex. Previous kinetics studies have shown that the dissociation of NADH is the rate-limiting step when ethanol is used as the substrate.

Overall, ADH relies on coenzyme dissociation and association that involves conformational changes and interconversion of NAD^{+}/NADH redox couple to catalyze the oxidation of alcohol to aldehyde.

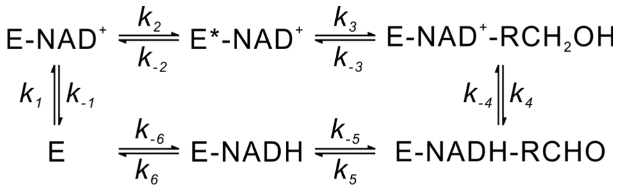

Supplementary Fig. 34 The mechanism of NAD^{+}-dependent ADH enzyme for the oxidation of alcohol to aldehyde[5].

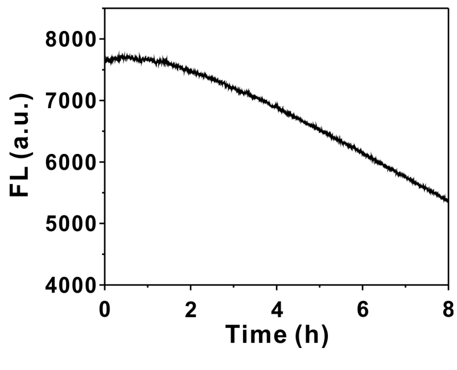

Supplementary Fig. 35 Time-dependent fluorescence at 330 nm as a result of the etching of ZIF-67 in Pro@ZIF-8@ADH/NAD^{+}@ZIF-67@ZIF-8. Excitation wavelength: 280 nm.

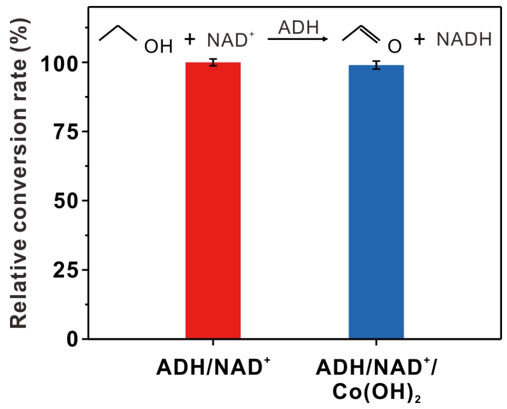

Supplementary Fig. 36 Catalytic efficiencies of ADH/NAD$^+$ in the absence (red) and in the presence (blue) of cobalt hydroxide. Note that the concentration of cobalt hydroxide was 1.1 mg mL^{-1}, which was calculated by assuming that ZIF-67 in Pro@ZIF-8@ADH/NAD$^+$@ZIF-67@ZIF-8 was completely dissociated; the concentrations of ADH and NAD$^+$ were 19.3 μg mL^{-1} and 17.1 μg mL^{-1}, which was the same with respective enzyme concentrations in all control experiments of Pro-ADH/NAD$^+$ cascade.

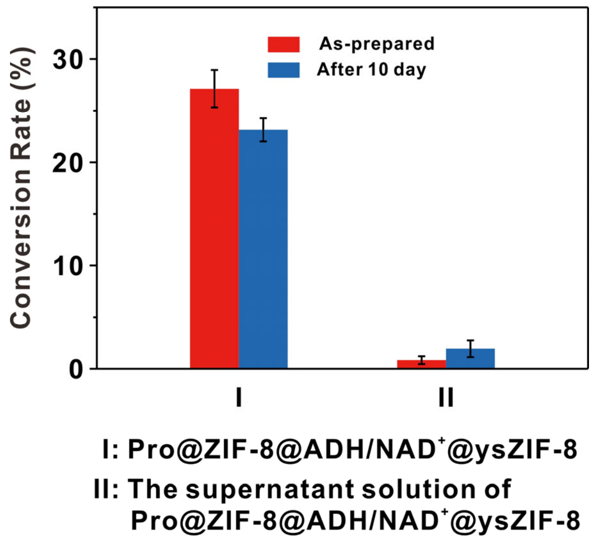

Supplementary Fig. 37 Comparison of long-term stability. Cascade activities of the Pro@ZIF-8@ADH/NAD$^+$@ysZIF-8 and supernatant solution of Pro@ZIF-8@ADH/NAD$^+$@ysZIF-8 immediately after fresh preparation (red) and after storage at room temperature for a time-interval of 10 day (blue), respectively.

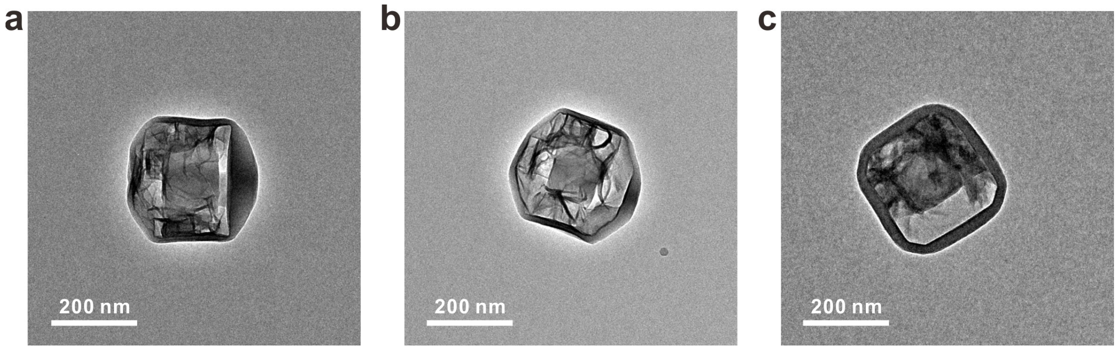

Supplementary Fig. 38 TEM images of Pro@ZIF-8@ADH/NAD^{+}@ysZIF-8 a) immediately after preparation, b) after storing at 4 °C for 10 day, and c) after catalytic reaction pos-storage, respectively.

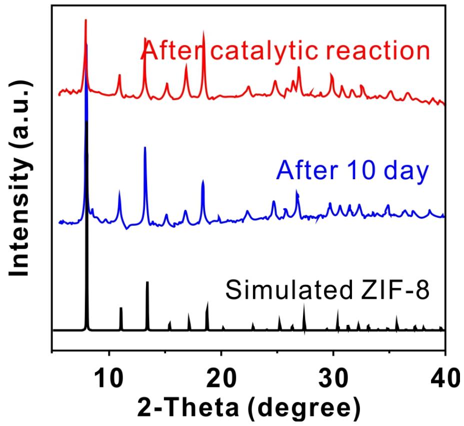

Supplementary Fig. 39 XRD pattern simulated from CIF file of ZIF-8 (black), XRD patterns of the Pro@ZIF-8@ADH/NAD^{+}@ysZIF-8 after storing at 4 °C for 10 day (blue), and after catalytic reaction post-storage (red), respectively.

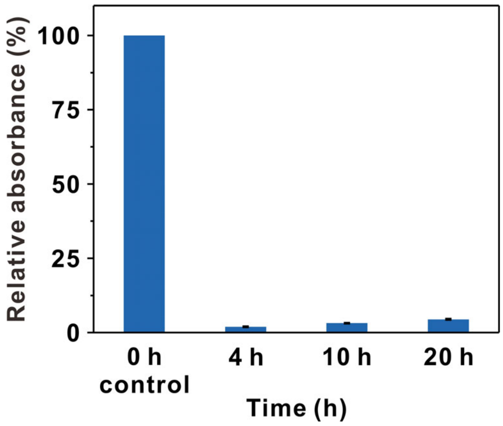

Supplementary Fig. 40 Leaching test of Pro@ZIF-8@ADH/NAD^{+}@ysZIF-8 at 4 h, 10h and 20 h. The amount of leaching enzymes was determined by Bradford assay. The 100% standard solution at 0 minute was tested against 5.35 μg mL^{-1} Pro, 19.3 μg mL^{-1} ADH and 17.1 μg mL^{-1} NAD^{+} in water.

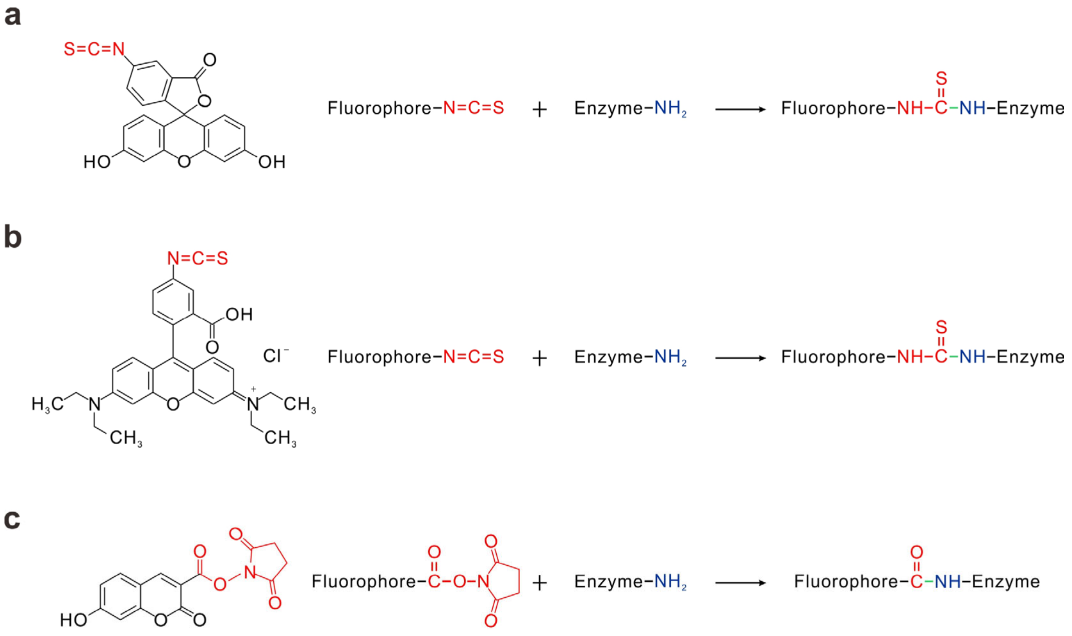

Supplementary Fig. 41 The amine-reactive fluorophores are acylating reagents that form thioureas or carboxamides upon reaction with amino groups of enzymes. a) Reaction of a primary amine with an isothiocyanate in FITC. b) Reaction of a primary amine with an isothiocyanate in Rhodamine B isothiocyanate. c) Reaction of a primary amine with a succinimidyl ester in 7-hydroxycoumarin-3-carboxylic acid N-succinimidyl ester.

Supplementary Tables

Supplementary Table 1. Summary of encapsulation methods and enzyme activities of GOx-HRP@MOF cascade catalytic systems.

GOx/HRP/MOF cascade catalytic systems	Encapsulation method	Application	Activity assay	Performance	References
GOx@ZIF-8@HRP@ZIF-8	Stepwise encapsulation of GOx and HRP by epitaxial shell-by-shell overgrowth	Biocatalytic cascades	Glucose + OPD	1) Compared to mixture of unassembled single-enzyme-loaded ZIF-8, 9.1-fold activity improvement; 2) Compared to mixture of free enzymes, 5.8-fold activity improvement	This work
GOx&HRP@ZIF-8	one-pot encapsulation of both GOx and HRP into ZIF-8	Biocatalytic cascades	Glucose + OPD	1) Compared to mixture of unassembled single-enzyme-loaded ZIF-8, 10.0-fold activity improvement; 2) Compared to mixture of free enzymes, 6.9-fold activity improvement	Control in this work
GOx&HRP@ZIF-8	one-pot encapsulation of both GOx and HRP into ZIF-8	Glucose biosensor	Glucose + $ABTS^{2-}$	1) Compared to mixture of unassembled single-enzyme-loaded ZIF-8, ~3-fold activity improvement; 2) Compared to mixture of free enzymes, comparable activity (~0.8-fold activity)	7
GOx&HRP@PCN-888	stepwise encapsulation of enzymes into the largest (accommodate GOx) and medium cages (accommodate HRP) of hierarchical PCN-888	Glucose detection	Glucose + $ABTS^{2-}$	1) NA; 2) Compared to mixture of free enzymes, 1.24-fold activity improvement	8
GOx&HRP@ZIF-8	one-pot encapsulation of both GOx and HRP into ZIF-8	Biocatalytic cascades	Glucose + Amplex Red	1) NA; 2) Compared to mixture of free enzymes, 7.5-fold activity improvement	9, 10
Diffusional mixture of GOx@UiO-66-capsules and HRP@UiO-66-capsules	separate encapsulation of GOx and HRP into UiO-66-capsules via water-in-oil Pickering emulsions	Segregate incompatible species	Glucose + $ABTS^{2-}$	NA; improvement in recyclability	11
Chains of GOx/ZIF-8A and HRP/ZIF-8A	complementary coiled-coil forming peptide induced assembly of enzyme encapsulated ZIF-8A (ZIF-8A: 3-amino-1,2,4-triazole (Atz) functionalized ZIF-8 to enable further peptide functionalization)	Stimuli-responsive biocatalytic cascades	Glucose + $ABTS^{2-}$	1) Compared to mixture of unassembled single-enzyme-loaded ZIF-8, 7.3-fold activity improvement; 2) NA	12
Diffusional mixture of GOx@ZIF-L-capsules and HRP@ZIF-L-capsules	separate encapsulation of GOx and HRP into ZIF-L-capsules via water-in-oil emulsions followed by metal-phenolic networks (MPNs) coating	Artificial cells	Glucose + $ABTS^{2-}$	Improvement in the stability upon ultraviolet irradiation, thermal treatment, and proteolysis	13, 14
GOx&HRP@ZIF-8	amino-acid-boosted one-pot encapsulation of both GOx and HRP into ZIF-8	Glucose biosensor	Glucose + TMB	1) NA; 2) Compared to mixture of free enzymes, ~3.2-fold activity improvement	15
GOx&HRP@ZIF-8	one-pot encapsulation of both GOx and HRP into ZIF-8	Starvation/oxidation therapy	Glucose + TMB	1) NA; 2) Compared to mixture of free enzymes, 3-fold activity improvement	16
GOx&HRP@DNA/ZIF-8	DNA scaffold cross-linking GOx and HRP, followed by one-pot encapsulation	Glucose biosensor	Glucose + $ABTS^{2-}$	1) NA; 2) Compared to mixture of free enzymes, 1.25-fold activity improvement	17

	into ZIF-8				
HRP@H-ZIF-8-GOx	HRP-immobilized hydrogel fibers as sacrificial template for hollow ZIF-8 encapsulating GOx	Biocatalytic cascades	Glucose + $ABTS^{2-}$	1) NA; 2) Compared to mixture of free enzymes, 3-fold activity improvement	18
GOx&HRP-A@ZIF-8	one-pot encapsulation of both GOx and acetic anhydride modified HRP (HRP-A) into ZIF-8	Glucose biosensor	Glucose + TMB	1) NA; 2) Compared to mixture of free enzymes, comparable activity (~0.8-fold activity)	19

Supplementary Table 2. The encapsulation efficiencies of enzymes in different systems. (E_0 refers that there is no enzyme.)

Enzyme	E@ZIF-8@E@ZIF-8	Encapsulation efficiencies
**GOx-FITC**	**GOx-FITC**@ZIF-8@E_0@ZIF-8	81.34%
	HRP@ZIF-8@**GOx-FITC**@ZIF-8	85.97%
**HRP-RhB**	**HRP-RhB**@ZIF-8@E_0@ZIF-8	69.06%
	GOx@ZIF-8@**HRP-RhB**@ZIF-8	49.65%
	GOx@ZIF-8^2@**HRP-RhB**@ZIF-8	53.01%
	GOx@ZIF-8^3@**HRP-RhB**@ZIF-8	58.62%
**Pro-RhB**	**Pro-RhB**@ZIF-8@E_0@ysZIF-8	77.38%
	ADH/NAD$^+$@ZIF-8@**Pro-RhB**@ysZIF-8	87.05%
**ADH-FITC**	**ADH-FITC**@ZIF-8@E_0@ysZIF-8	87.05%
	Pro@ZIF-8@**ADH-FITC**@ysZIF-8	55.20%
	Pro@ZIF-8^2@**ADH-FITC**@ysZIF-8	59.33%
	Pro@ZIF-8^3@**ADH-FITC**@ysZIF-8	63.13%
**NAD$^+$-coumarin**	**NAD$^+$-coumarin**@ZIF-8@E_0@ysZIF-8	41.87%
	Pro@ZIF-8@**NAD$^+$-coumarin**@ysZIF-8	48.90%
	Pro@ZIF-8^2@**NAD$^+$-coumarin**@ysZIF-8	53.71%
	Pro@ZIF-8^3@**NAD$^+$-coumarin**@ysZIF-8	60.29%

Note that the differences in encapsulation efficiencies of multi-enzymes might be influenced by various factors, including enzyme-MOF interactions and loading spaces.[10]

Supplementary Table 3. Weight loss in ZIF-8, pure enzymes, and GOx@ZIF-8@HRP@ZIF-8.

	**Weight at 25 °C**	**Weight at 800 °C**	**Weight loss from 25 °C to 800 °C**
GOx	100%	15.5%	84.5%
HRP	100%	19.9%	80.1%
ZIF-8	100%	37.5%	62.5%
GOx@ZIF-8@HRP@ZIF-8	100%	27.6%	72.4%

On the basis of enzyme loading characterization results and the final product weight, we determined the loadings of GOx and HRP to be 70.72 and 215.9 μg mg^{-1}; and weight percentage of GOx, HRP, and ZIF-8 in the final product to be 7.1 wt%, 21.6 wt%, and 71.3 wt%. Through the following equation, the weight loss in GOx@ZIF-8@HRP@ZIF-8 can be estimated as 67.9 wt%, which is similar to the corresponding measured weight loss in TGA curve (72.4 wt%). Based on the above results, we can conclude that the weight loss in TGA matches with the enzymes loading.

$$\text{weight loss } X_{\text{GOx@ZIF-8@HRP@ZIF-8}}(\%) = m_{\text{GOx}} \times X_{\text{GOx}} + m_{\text{HRP}} \times X_{\text{HRP}} + m_{\text{ZIF-8}} \times X_{\text{ZIF-8}}$$
$$= 7.1\% \times 84.5\% + 21.6\% \times 80.1\% + 71.3\% \times 62.5\% = 67.9\%$$

m: weight percentage of each component; X: weight loss of each component.

Supplementary Table 4. Weight loss in ZIF-8@ysZIF-8, pure enzymes, and Pro@ZIF-8@ADH/NAD$^+$@ysZIF-8.

	Weight at 25 °C	Weight at 800 °C	Weight loss from 25 °C to 800 °C
Pro	100%	12.5%	87.5%
ADH	100%	18.5%	81.5%
NAD$^+$	100%	35.6%	64.4%
ZIF-8@ysZIF-8	100%	21.6%	78.4%
Pro@ZIF-8@ADH/NAD$^+$@ysZIF-8	100%	27.6%	72.4%

On the basis of enzyme loading characterization results and the final product weight, we determined the loadings of Pro, ADH, and NAD$^+$ to be 53.49, 193.0, and 170.9 μg mg^{-1}; weight percentage of Pro, ADH, NAD$^+$, and ZIF-8@ysZIF-8 in the final product to be 5.6 wt%, 19.3 wt%, 17.1 wt%, and 58.2 wt%. Through the following equation, the weight loss in Pro@ZIF-8@ADH/NAD$^+$@ysZIF-8 can be estimated as 77.3 wt%, which is similar to the corresponding measured weight loss in TGA curve (72.4 wt%). Based on the above results, we can conclude that the weight loss in TGA matches with the enzymes loading.

$$\begin{aligned}\text{weight loss } X_{\text{Pro@ZIF-8@ADH/NAD}^+\text{@ysZIF-8}}(\%) &= m_{\text{Pro}} \times X_{\text{Pro}} + m_{\text{ADH}} \times X_{\text{ADH}} \\ &+ m_{\text{NAD}^+} \times X_{\text{NAD}^+} + m_{\text{ZIF-8@ysZIF-8}} \times X_{\text{ZIF-8@ysZIF-8}} \\ &= 5.6\% \times 87.5\% + 19.3\% \times 81.5\% + 17.1\% \times 64.4\% + 58.2\% \times 78.4\% \\ &= 77.3\%\end{aligned}$$

Supplementary Table 5. The size of GOx@ZIF-8, GOx@ZIF-8[2], and GOx@ZIF-8[3], respectively.

	GOx@ZIF-8	GOx@ZIF-8[2]	GOx@ZIF-8[3]
size of the particle	~120 nm	~160 nm	~240 nm

Supplementary Table 6. The size of Pro@ZIF-8, Pro@ZIF-8[2], and Pro@ZIF-8[3], respectively.

	Pro@ZIF-8	Pro@ZIF-8[2]	Pro@ZIF-8[3]
size of the particle	~140 nm	~180 nm	~260 nm

Supplementary Table 7. The amounts of enzymes added in the synthetic protocol for different systems.

E@ZIF-8	Enzyme loading	The initially added enzyme amount
GOx@ZIF-8@HRP@ZIF-8	GOx: 70.72 μg mg^{-1}<br>HRP: 215.9 μg mg^{-1}	GOx: 3 mg, HRP: 3 mg
GOx@ZIF-8^2@HRP@ZIF-8	GOx: 65.95 μg mg^{-1}<br>HRP: 200.6 μg mg^{-1}	GOx: 3 mg, HRP: 2.8 mg
GOx@ZIF-8^3@HRP@ZIF-8	GOx: 60.25 μg mg^{-1}<br>HRP: 180.9 μg mg^{-1}	GOx: 3 mg, HRP: 2.5 mg
HRP@ZIF-8@GOx@ZIF-8	HRP: 203.0 μg mg^{-1}<br>GOx: 68.93 μg mg^{-1}	HRP: 11 mg, GOx: 0.6 mg
GOx@ZIF-8<br>HRP@ZIF-8	GOx: 65.08 μg mg^{-1}<br>HRP: 219.5 μg mg^{-1}	GOx: 0.6 mg<br>HRP: 2.2 mg
Gox/HRP@ZIF-8	GOx: 68.74 μg mg^{-1}<br>HRP: 213.9 μg mg^{-1}	GOx: 0.6 mg, HRP: 2.2 mg
Pro@ZIF-8@ADH/NAD^+@ysZIF-8	Pro: 53.49 μg mg^{-1}<br>ADH: 193.0 μg mg^{-1}<br>NAD^+: 170.9 μg mg^{-1}	Pro: 3 mg, ADH: 3 mg, NAD^+: 3 mg
Pro@ZIF-8^2@ADH/NAD^+@ysZIF-8	Pro: 51.58 μg mg^{-1}<br>ADH: 184.6 μg mg^{-1}<br>NAD^+: 161.1 μg mg^{-1}	Pro: 3 mg, ADH: 2.8 mg, NAD^+: 2.7 mg
Pro@ZIF-8^3@ADH/NAD^+@ysZIF-8	Pro: 48.87 μg mg^{-1}<br>ADH: 182.4 μg mg^{-1}<br>NAD^+: 152.3 μg mg^{-1}	Pro: 3 mg, ADH: 2.6 mg, NAD^+: 2.4 mg
ADH/NAD^+@ysZIF-8@Pro@ZIF-8	ADH: 174.1 μg mg^{-1}<br>NAD^+: 154.3 μg mg^{-1}<br>Pro: 54.98 μg mg^{-1}	ADH: 9.5 mg, NAD^+: 17.5 mg, Pro: 0.6 mg
Pro@ZIF-8<br>ADH/NAD^+@ysZIF-8	Pro: 55.27 μg mg^{-1}<br>ADH: 177.0 μg mg^{-1}<br>NAD^+: 168.4 μg mg^{-1}	Pro: 0.6 mg<br>ADH: 1.9 mg, NAD^+: 3.5 mg
Pro/ADH/NAD^+@ysZIF-8	Pro: 53.99 μg mg^{-1}<br>ADH: 192.3 μg mg^{-1}	Pro: 0.6 mg, ADH: 1.9 mg, NAD^+: 3.5 mg

	NAD^+: 170.4 μg mg^{-1}	
Pro@ZIF-8@ADH/NAD^+@ZIF-8	Pro: 54.62 μg mg^{-1}<br>ADH: 194.8 μg mg^{-1}<br>NAD^+: 172.6 μg mg^{-1}	Pro: 3 mg, ADH: 3 mg, NAD^+: 3 mg

Supplementary References

1. Huth, F. *et al*. Nano-FTIR absorption spectroscopy of molecular fingerprints at 20 nm spatial resolution. *Arch. Nano. Lett.* **12**, 3973-3978 (2012).

2. Govyadinov, A. A. *et al*. Quantitative measurement of local Infrared absorption and dielectric function with tip-enhanced near-field microscopy. *J. Phys. Chem. Lett.* **4**, 1526-1531 (2013).

3. Amenabar, I. *et al*. Structural analysis and mapping of individual protein complexes by infrared nanospectroscopy. *Nat. Commun.* **4**, 2890 (2013).

4. Plapp, B. V. Conformational changes and catalysis by alcohol dehydrogenase. *Arch. Biochem. Biophys.* **493**, 3-12 (2010).

5. LeBrun, L. A. & Plapp, B. V. Control of coenzyme binding to horse liver alcohol dehydrogenase. *Biochemistry* **38**, 12387-12393 (1999).

6. Kim, K. & Plapp, B. V. Substitution of cysteine-153 ligated to the catalytic zinc in yeast alcohol dehydrogenase with aspartic acid and analysis of mechanisms of related medium chain dehydrogenases. *Chem. Bio. Interact.* **302**, 172-182 (2019).

7. Wu, X. L., Ge, J., Yang, C., Hou, M. & Liu, Z. Facile synthesis of multiple enzyme-containing metal–organic frameworks in a biomolecule-friendly environment. *Chem. Commun.* **51**, 13408 (2015).

8. Lian, X. Z., Chen, Y. P., Liu, T. F. & Zhou, H. C. Coupling two enzymes into a tandem nanoreactor utilizing a hierarchically structured MOF. *Chem. Sci.* **7**, 6969 (2016).

9. Chen, W. H., Vázquez-González, M., Zoabi, A., AbuReziq, R. & Willner, I. Biocatalytic cascades driven by enzymes encapsulated in metal–organic framework nanoparticles. *Nat. Cat.* **1**, 689-695 (2018).

10. Vázquez-González, M., Wang, C. & Willner, I. Biocatalytic cascades operating on macromolecular scaffolds and in confined environments. *Nat. Cat.* **3**, 256-273 (2020).

11. Xu, Z. *et al.* Compartmentalization within Self-assembled metal-organic framework nanoparticles for tandem reactions. *Adv. Funct. Mater.* **28**, 1802479 (2018).

12. Liang, J. *et al.* Peptide-induced super-assembly of biocatalytic metal-organic frameworks for programmed enzyme cascades. *Chem. Sci.* **10**, 7852-7858 (2019).

13. Liu, J., Guo, Z. Y. & Liang, K. Biocatalytic metal-organic framework-based artificial cells. *Adv. Funct. Mater.* **29**, 1905321 (2019).

14. Liang, J. Y. & Liang, K. Multi-enzyme cascade reactions in metal-organic frameworks. *Chem. Rec.* **20**, 1100-1116 (2020).

15. Chen, G. S. *et al.* A convenient and versatile amino-acid-boosted biomimetic strategy for the nondestructive encapsulation of biomacromolecules within metal-organic frameworks. *Angew. Chem. Int. Ed.* **58**, 1463-1467 (2019).

16. Bai, J., Peng, C. J., Guo, L. P. & Zhou, M. Metal-organicframework-integrated enzymes as bioreactor for enhanced therapy against solid tumor via a cascade catalytic reaction. *ACS Biomater. Sci. Eng.* **5**, 6207-6215 (2019).

17. Song, J. Y. *et al*. Construction of multiple enzyme metal-organic frameworks biocatalyst via DNA scaffold: A promising strategy for enzyme encapsulation. *Chem. Eng. J.* **363**, 174-182 (2019).

18. Liu, H. J. *et al*. Compartmentalization of biocatalysts by immobilizing bienzyme in hollow ZIF-8 for colorimetric detection of glucose and phenol. *Ind. Eng. Chem. Res.* **59**, 42-51 (2020).

19. Chen, G. S. *et al*. Modulating the biofunctionality of metal-organic-framework encapsulated enzymes through controllable embedding patterns. *Angew. Chem. Int. Ed.* **132**, 2889-2896 (2020).
